# Cyclin A2/E1 activation defines a hepatocellular carcinoma subclass with a rearrangement signature of replication stress

**DOI:** 10.1038/s41467-018-07552-9

**Published:** 2018-12-07

**Authors:** Quentin Bayard, Léa Meunier, Camille Peneau, Victor Renault, Jayendra Shinde, Jean-Charles Nault, Iadh Mami, Gabrielle Couchy, Giuliana Amaddeo, Emmanuel Tubacher, Delphine Bacq, Vincent Meyer, Tiziana La Bella, Audrey Debaillon-Vesque, Paulette Bioulac-Sage, Olivier Seror, Jean-Frédéric Blanc, Julien Calderaro, Jean-François Deleuze, Sandrine Imbeaud, Jessica Zucman-Rossi, Eric Letouzé

**Affiliations:** 1 INSERM, UMR-1162, Génomique Fonctionnelle des Tumeurs Solides, Equipe Labellisée Ligue Contre le Cancer, Institut Universitaire d’Hématologie, Paris, 75010 France; 2 Université Paris Descartes, Labex Immuno-Oncology, Sorbonne Paris Cité, Faculté de Médecine, Paris, 75006 France; 3 Université Paris 13, Sorbonne Paris Cité, Unité de Formation et de Recherche Santé, Médecine, Biologie Humaine, Bobigny, 93017 France; 4 Université Paris Diderot, Sorbonne Paris Cité, Paris, 75013 France; 5 Laboratory for Bioinformatics, Fondation Jean Dausset – CEPH, Paris, 75010 France; 6 Liver unit, Hôpital Jean Verdier, Hôpitaux Universitaires Paris-Seine-Saint-Denis, Assistance-Publique Hôpitaux de Paris, APHP, Bondy, 93140 France; 7 Unité de Formation et de Recherche Santé Médecine et Biologie Humaine, Université Paris 13, Communauté d’Universités et Etablissements Sorbonne Paris Cité, Bobigny, 93017 France; 8 Inserm, U955, Team 18, Université Paris-Est Créteil, Faculté de Médecine, Créteil, 94010 France; 9 Assistance Publique-Hôpitaux de Paris, Service d’Hépatologie, CHU Henri Mondor, Créteil, 94010 France; 10 Centre National de Recherche en Génomique Humaine, CEA, Evry, 91000 France; 11 Service Hépato-Gastroentérologie et Oncologie Digestive, Hôpital Haut-Lévêque, Centre Hospitalier Universitaire de Bordeaux, Bordeaux, 33076 France; 12 Université Bordeaux, Bordeaux Research in Translational Oncology, Bordeaux, 33076 France; 13 Service de Pathologie, Hôpital Pellegrin, Centre Hospitalier Universitaire de Bordeaux, Bordeaux, 33000 France; 14 Radiology Department, Jean Verdier Hospital, Hôpitaux Universitaires Paris-Seine-Saint-Denis, APHP, Bondy, 93140 France; 15 Assistance Publique-Hôpitaux de Paris, Département de Pathologie, Hôpital Henri Mondor, Créteil, 94010 France; 16 Assistance Publique-Hôpitaux de Paris, Hopital Européen Georges Pompidou, 75015 Paris, France

## Abstract

Cyclins A2 and E1 regulate the cell cycle by promoting S phase entry and progression. Here, we identify a hepatocellular carcinoma (HCC) subgroup exhibiting cyclin activation through various mechanisms including hepatitis B virus (HBV) and adeno-associated virus type 2 (AAV2) insertions, enhancer hijacking and recurrent *CCNA2* fusions. Cyclin A2 or E1 alterations define a homogenous entity of aggressive HCC, mostly developed in non-cirrhotic patients, characterized by a transcriptional activation of E2F and ATR pathways and a high frequency of *RB1* and *PTEN* inactivation. Cyclin-driven HCC display a unique signature of structural rearrangements with hundreds of tandem duplications and templated insertions frequently activating *TERT* promoter. These rearrangements, strongly enriched in early-replicated active chromatin regions, are consistent with a break-induced replication mechanism. Pan-cancer analysis reveals a similar signature in *BRCA1*-mutated breast and ovarian cancers. Together, this analysis reveals a new poor prognosis HCC entity and a rearrangement signature related to replication stress.

## Abstract (web summary)

Cyclins A2 and E1 are known to regulate the cell cycle by promoting S phase entry and progression. Here, they identify an aggressive hepatocellular carcinoma subgroup exhibiting cyclin activation through various mechanisms and find this subgroup to display replication stress-induced structural rearrangements frequently activating TERT promoter.

## Introduction

Hepatocellular carcinoma (HCC) is the third leading cause of cancer death worldwide. Only 30% of cases are diagnosed at an early stage and are amenable to curative treatment by tumor resection or liver transplantation^1^. The multikinase inhibitors sorafenib^2^ and regorafenib^3^ are currently the only drugs approved for advanced HCC cases, but the median life expectancy of patients with HCC on sorafenib is only 1 year. All phase III clinical trials involving targeted molecular therapies have failed so far for various reasons including liver toxicity, lack of antitumoral potency, and the molecular heterogeneity of the disease^4^. Identifying homogeneous HCC subgroups sharing similar driving mechanisms and vulnerabilities is thus crucial to design successful patient-tailored clinical trials.

Most HCC develop in a cirrhotic liver, associated with various etiologies including hepatitis B virus (HBV) and hepatitis C virus (HCV) infections, alcohol abuse, metabolic disease, and exposure to carcinogenic compounds like aflatoxin B1^5^. The natural history of HCC in cirrhosis follows a well-established sequence with the successive development of dysplastic nodules that can transform into early stage and advanced HCC. *TERT* promoter mutations are the initial oncogenic events already detected in dysplastic nodules^6^ whereas alterations in other HCC drivers^7–11^ involved in cell cycle control (*TP53*, *RB1*, *CCND1*, *CDKN2A*), Wnt/ß-catenin signaling (*CTNNB1*, *AXIN1*), oxidative stress response (*NFE2L2*, *KEAP1*) epigenetic regulation (*ARID1A*, *ARID2*) and the AKT/mTOR and MAP kinase pathway (*RPS6KA3*, *TSC1*, *TSC2*, *PTEN*) only occur in progressed HCC^12^.

In 20% of the cases, HCC develops in absence of cirrhosis. These patients usually maintain adequate liver functions and, being less subject to liver toxicity, may be eligible for more treatment options. The etiology of HCC in absence of cirrhosis is largely unknown, but one mechanism of transformation involves insertional mutagenesis by the HBV virus. The first oncogenic HBV insertion was identified in cyclin A2 gene (*CCNA2*)^13^. Since then, recurrent HBV insertions were mapped in several oncogenes including *CCNE1*, *KMT2B* and *TERT*^14,15^. Recently, we identified adeno-associated virus type 2 (AAV2) insertions as a new etiology for HCC developed in absence of cirrhosis, with recurrent insertions in *CCNA2* and *CCNE1* genes^16^. However, the molecular consequences of viral insertions in cyclin genes and their precise role in HCC development remain poorly understood.

Here, we report the systematic screening of *CCNA2* and *CCNE1* alterations in 751 HCC. We identify new mechanisms of cyclin A2/E1 activation, and we explore the clinical and molecular characteristics of this tumor subgroup.

## Results

### Viral insertions and gene fusions activate cyclin A2

To identify the exhaustive landscape of *CCNA2* and *CCNE1* alterations in HCC, we analyzed 751 HCC comprising an in-house series of 160 tumors (LICA-FR) analyzed by RNA sequencing (RNAseq, *n* = 160), whole exome (WES, *n* = 156) and whole genome sequencing (WGS, *n* = 45) (Supplementary Data 1), the TCGA^17^ series (334 HCC with RNA-seq and WES, 48 or which also analyzed by WGS) and the ICGC-JP^11^ series (257 HCC with WGS data, Supplementary Data 2).

**Fig. 1:**
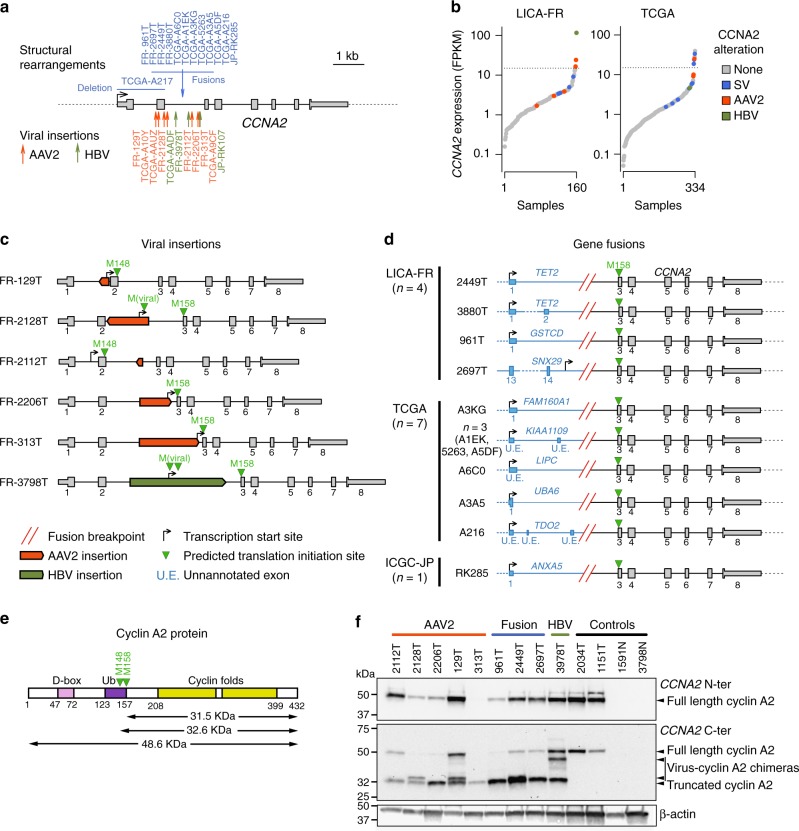
Diverse mechanisms leading to *CCNA2* activation in HCC. **a** Summary of structural rearrangements (top) and viral insertions (bottom) affecting *CCNA2* gene identified in 751 HCC from the LICA-FR, TCGA and ICGC-JP cohorts. **b** Sorted *CCNA2* expression (log scale) in the LICA-FR and TCGA cohorts. Gene expression was obtained from RNA-seq data and is given in fragments per kilobase of exons per million reads (FPKM). Samples harboring structural variants (SV) or viral insertions are indicated with a color code. **c** Functional consequences of AAV2 and HBV insertions in *CCNA2*. Viral insertions identified in the LICA-FR cohort were precisely mapped using WGS or viral capture data, and RNA-seq reads were aligned on the reconstructed chimeric DNA to identify the transcription start sites and predicted translation initiation sites of abnormal transcripts. **d** *CCNA2* fusions identified in the LICA-FR, TCGA and ICGC-JP cohorts. The transcription start site of the fusion transcript is represented together with the predicted translation initiation site. Fusions with *KIAA1109*, *LIPC* and *TDO2* involve 5′ exons not annotated in transcript databases but expressed in normal liver. **e** Schematic representation of cyclin A2 protein with functional domains. D-box Destruction box; Ub, Ubiquitination targeting sequences. **f** Western blot analysis of cyclin A2 using antibodies targeting the N-terminal (top) or C-terminal (middle) domains. Tumors with viral insertions or gene fusions are compared with tumors without *CCNA2* alteration and non-tumoral liver controls

We first screened the LICA-FR series of 160 tumors to characterize the exhaustive mechanisms activating *CCNA2* and *CCNE1* in HCC. We identified one HBV and 5 AAV2 insertions (four previously described in the ref. ^16^) in *CCNA2* gene (Supplementary Data 3), all but one located within *CCNA2* intron 2 (Fig. 1a). Viral insertions were associated with *CCNA2* mRNA over-expression (*P* = 8.2 × 10^−9^, fold-change = 5.6, Fig. 1b), but also altered the transcript and protein structure. AAV2 and HBV insertions induced the expression of various abnormal transcripts (Supplementary Fig. 1), predicted to generate a truncated cyclin A2 protein starting at methionine 148 or 158 with occasionally a few amino acids translated from the viral genome (Fig. 1c).

In addition we identified novel gene fusions in 4 tumors (Supplementary Data 4), all involving the C-terminal part of *CCNA2* (exons 3–8) at chromosome 4q27 downstream 3 different partner genes: *GSTCD* at 4q24, *SNX29* at 16p13.13 and *TET2* (×2) at 4q24 (Fig. 1a, d). In the *TET2-CCNA2* and *GSTCD-CCNA2* fusion transcripts, the first untranslated exons of *TET2* and *GSTCD* were linked with *CCNA2* exons 3–8. The *SNX29-CCNA2* fusion revealed an alternative transcription start site (TSS) in *SNX29* intron 14 generating a 448-nucleotide sequence spliced with *CCNA2* exon 3. In all fusions, the predicted translation initiation site of the fused RNA was located at methionine 158 in *CCNA2* exon 3, predicted to generate a truncated cyclin A2 protein of 275 amino acids (32 Kda), lacking the destruction box^18^ and ubiquitination targeting sequences^19^ but retaining the functional cyclin box, without any protein fragment from the partner genes (Fig. 1e).

Western blot analysis of 9 tumors with viral insertion or gene fusion confirmed the over-expression, as predicted, of a truncated 32 KDa protein (Fig. 1f). Thus, gene fusions and viral insertions in *CCNA2* both lead to the production of a stable protein lacking the N-terminal regulatory domains.

In the TCGA series, we identified 7 *CCNA2* fusions with 5 different partner genes (*FAM160A1*, *KIAA1109* × 3, *LIPC*, *UBA6* and *TDO2*, Fig. 1a, d), all of which involved the first untranslated exon(s) of the partner gene linked with exons 3–8 of *CCNA2*. WGS revealed in another tumor a focal deletion starting in the 5′ UTR region and ending in *CCNA2* intron 2 (Supplementary Fig. 2). All these events were predicted to generate the same 32 KDa truncated cyclin A2 protein lacking N-terminal regulatory domains. We also identified one tumor with HBV insertion and 3 tumors with AAV2 insertions in *CCNA2*. Finally, 6 tumors strongly overexpressed *CCNA2* (FPKM > 15), 3 of which displayed 23–48 Mb intra-chromosomal deletions linking the intergenic region downstream *CCNA2* with the highly expressed *ALB*, *AFP*, and *ADH6* genes (Supplementary Fig. 2). The ICGC-JP cohort comprised one HBV insertion in *CCNA2* intron 2 and one fusion between the first untranslated exon of *ANXA5* and exons 3–8 of *CCNA2* (Fig. 1a, d).

In total, we identified 10 HCC with *CCNA2* activation events in the LICA-FR series (6.2%), 2 in the ICGC-JP series (0.8%) and 18 in the TCGA series (5.4%), associated with a significant increase of *CCNA2* mRNA expression, but also generating a truncated cyclin A2 protein lacking the N-terminal destruction box and the ubiquitination site.

### Viral insertions and enhancer hijacking activate cyclin E1

**Fig. 2:**
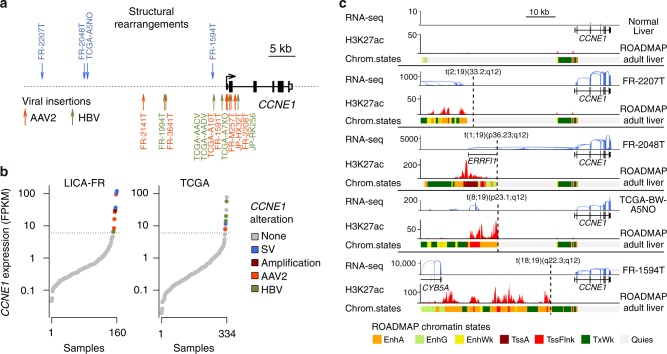
Viral and non-viral mechanisms of *CCNE1* activation in HCC. **a** Summary of structural rearrangements (top) and viral insertions (bottom) affecting *CCNE1* gene identified in 751 HCC from the LICA-FR, TCGA and ICGC-JP cohorts. **b** Sorted *CCNE1* expression (log scale) in the LICA-FR and TCGA cohorts. Gene expression was obtained from RNA-seq data and is given in fragments per kilobase of exons per million reads (FPKM). Samples harboring structural variants (SV), focal amplifications and viral insertions are indicated with a color code. **c** Functional consequences of structural rearrangements affecting *CCNE1* regulatory region. RNA-seq read counts along *CCNE1* locus are represented in normal liver (top) and in 4 tumors harboring structural rearrangements upstream *CCNE1* trancription start site (TSS). H3K27Ac chromatin immunoprecipitation sequencing (ChIP-seq) signal and chromatin states in adult liver were obtained from the ROADMAP consortium and are depicted below each reconstruted DNA sequence. EnhA: active enhancer; EngG: genic enhancer; EnhWk: weak enhancer; TssA: active TSS; TssFlnk: flanking TSS; TxWk: weak transcription; Quies: quiescent chromatin

In our series of 160 HCC, we identified 5 AAV2 insertions (three previously described in the ref. ^16^) and one HBV insertion in the 5′ region or upstream the transcription start site (TSS) of *CCNE1* (Fig. 2a, Supplementary Data 3). These viral insertions induced a massive overexpression of the full-length *CCNE1* gene (Fig. 2b), confirmed by western-blot analysis (Supplementary Fig. 3). Interestingly, one case with AAV2 insertion (FR2141T) also displayed an amplification of *CCNE1* locus including the viral sequence (Supplementary Fig. 3), suggesting a two-step selection of *CCNE1* activation in the natural history of this tumor. Four other tumors overexpressed *CCNE1* (FPKM > 6), explained by high-level amplification in one case. In the 3 remaining cases, whole genome sequencing revealed interchromosomal translocation breakpoints in the regulatory region of *CCNE1* (Fig. 2a). Tumor FR2048T displayed a translocation placing *CCNE1* downstream the first untranslated exon of the highly expressed *ERRFI1* gene, leading to a highly expressed *ERRFI1*-*CCNE1* fusion. The two other translocations lead to juxtapose *CCNE1* promoter with enhancer-rich chromatin areas located close to the highly expressed genes *RAPH1* and *CYB5A* (Fig. 2c). Thus, both viral insertions and structural rearrangements can activate *CCNE1* expression by bringing viral or distal human enhancers in the regulatory region of the gene.

In the TCGA series, 10 tumors overexpressed *CCNE1* (Fig. 2b), including 2 cases with HBV insertion, one with HBV insertion plus high-level amplification, one with AAV2 insertion and one with a translocation between *CCNE1* regulatory region and an enhancer-rich region on chromosome 5 (Fig. 2c). In the 5 remaining cases, the mechanism leading to *CCNE1* overexpression remained unexplained in absence of WGS data. In the ICGC-JP cohort, we identified one AAV2 and one HBV insertion associated with *CCNE1* overexpression. In total, we identified 10 HCC with *CCNE1* activation events in the LICA-FR cohort (6.2%), two in the ICGC series (0.8%) and 10 in the TCGA series (3.0%).

Across the three data sets, 52/751 tumors (6.9%) displayed an activation of cyclin A2 (*n* = 30) or E1 (*n* = 22) due to viral insertions or structural rearrangements. These are later referred to as CCN-HCC. The proportion of CCN-HCC varied between the cohorts (12.5% in our series, 8.4% in TCGA and 1.6% in ICGC-JP) due to differences in etiological backgrounds (Supplementary Data 2). It was particularly high in our series enriched in cancers developed in a non-fibrotic liver, and low in the IGGC-Japan series dominated by HCV-related cases.

### Cyclin A2 or E1 activation defines a homogenous HCC subgroup

**Fig. 3:**
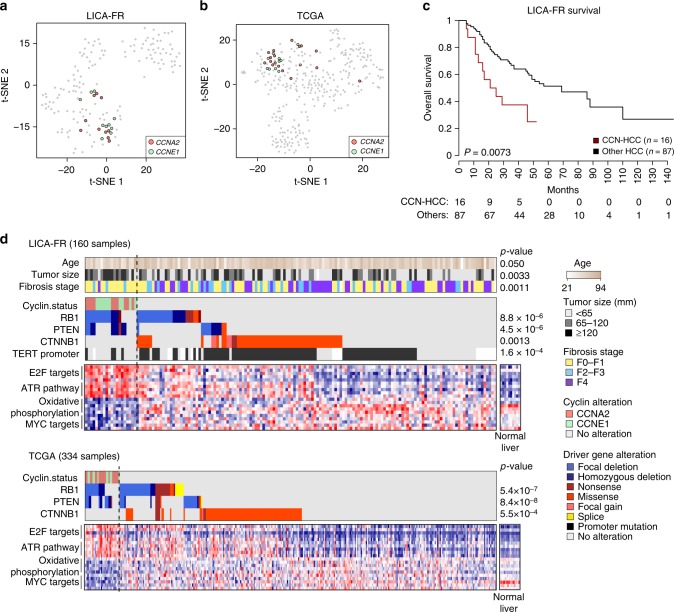
Clinical and molecular features of cyclin-activated HCC. **a** t-SNE plots depicting the classification of HCC from the LICA-FR and TCGA cohorts based on their transcriptional profiles. Tumors harboring *CCNA2* or *CCNE1* activating alterations are indicated with a color code. **b** Clinical characteristics, driver genes and deregulated pathways associated with CCN-HCC in the LICA-FR (top) and TCGA (bottom) cohorts. **c** Overall survival in CCN-HCC as compared with other HCC in the LICA-FR cohort. Only HCC with curative resection (R0) were included

We next explored the molecular and clinical characteristics of CCN-HCC. Gene expression analysis of the LICA-FR and TCGA showed that CCN-HCC defined homogeneous transcriptional clusters (Fig. 3a). They were characterized by an overexpression of cell cycle genes, in particular E2F targets, and an activation of the ATR pathway in response to replication stress (Fig. 3b, Supplementary Data 5). The most significant down-regulated pathways were oxidative phosphorylation, suggesting a metabolic switch to aerobic glycolysis (Warburg effect), and *MYC* targets. We also compared the alteration frequencies of known liver cancer driver genes^10^ between CCN-HCC and others. *CCNA2* and *CCNE1* activation events were remarkably exclusive from *CTNNB1* and *TERT* promoter mutations, but frequently associated with *PTEN* and *RB1* inactivation in both the LICA-FR and TCGA series (Fig. 3b, Supplementary Data 6). *RB1* inactivation may allow cells to overcome oncogene-induced senescence^20^ in these tumors, whereas *PTEN* inactivation might favor the oncogenic metabolic switch that we observed at the transcriptional level^21^. Compared to the other tumors in the LICA-FR series, CCN-HCC were enriched in large tumors (median largest nodule diameter = 115 vs. 60 mm, *P* = 0.0033), of poor prognosis (median overall survival = 21 vs. 69 months, *P* = 0.0072, Fig. 3c), developed in younger patients (median age = 57 vs. 67 years old, *P* = 0.050) with a non-fibrotic liver (fibrosis stage F0-F1 80 vs. 42%, *P* = 0.0011). Thus, CCN-HCC define a homogenous HCC entity with characteristic clinical and molecular features.

### CCN-HCC display a unique structural rearrangement signature

To identify mutational signatures associated with CCN-HCC, we analyzed the whole genome sequences of 45 of our 160 HCC (35 were previously published^22^, 10 new), including 13 CCN-HCC. With a median of 12,463 mutations, CCN-HCC were rather less mutated than others (median = 16,397 mutations, *P* = 0.065). Mutational signatures 4, 5, and 16 (COSMIC nomenclature), ubiquitous in liver cancers^22^, accounted for most mutations in CCN-HCC, with a slight increase of signature 5 (53 vs. 33%, *P* = 0.036) and decrease of signature 16 (23 vs. 32%, *P* = 0.05) as compared with other HCC (Supplementary Fig. 4).

**Fig. 4:**
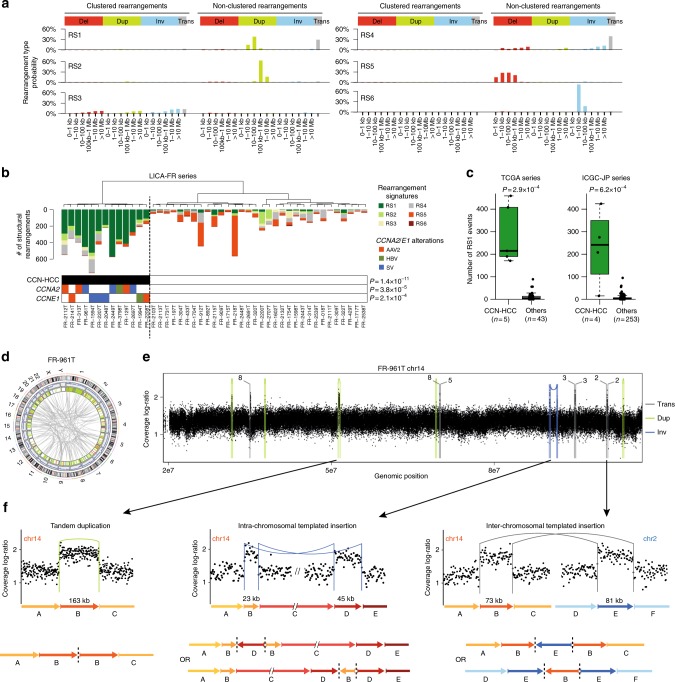
Cyclin-activated HCC display a specific signature or structural rearrangements. **a** Six rearrangement signatures identified across 350 HCC genomes by non-negative matrix factorization. Structural rearrangements were classified in 38 categories considering their type (del: deletion, dup: tandem duplication, inv: inversion, trans: inter-chromosomal translocation) and size, and distinguishing clustered from non-clustered events. The probability of each rearrangement category in each signature is represented, with rearrangement types indicated above and rearrangement sizes below. **b** Unsupervised classification of 45 HCC from the LICA-FR cohort based on the contribution of rearrangement signatures in each tumor. Significant molecular alterations associated with the cluster of tumors having a high contribution of signature RS1 are represented below. *P*-values were obtained using Fisher’s exact tests. **c** Validation of the association between the RS1 signature and CCN-HCC in the TCGA and ICGC-JP series. The middle bar, median; box, interquartile range; bars extend to 1.5 times the interquartile range. **d** CIRCOS plot representing the structural rearrangement profile of a representative CCN-HCC (FR-961T, harboring a *GSTCD*-*CCNA2* fusion). **e** Copy-number profile showing the accumulation of focal gains along chromosome 14 in tumor FR-961T. Structural rearrangements are overlaid on the copy-number profile with a color code indicating the type of event. trans: inter-chromosomal translocation; dup: tandem duplication; inv: inversion. **f** Three types of rearrangements leading to focal chromosome gains in CCN-HCC. A representative example of each type of event is shown with a copy-number plot above and a schematic representation of the rearranged chromosome below. Structural rearrangements are represented with the same color code as in **e**. Dashed lines on schematic chromosome reconstructions represent the abnormal junctions detected in WGS data

In contrast, CCN-HCC displayed > 3 times more structural variants (median = 415 vs. 126, *P* = 1.1 × 10^−4^). We identified 6 rearrangement signatures, termed RS1 to RS6, characterized by different combinations of rearrangement categories defined according to the type, size, and clustered nature of rearrangements (Fig. 4a). Strikingly, a high number of rearrangements attributed to signature RS1 (≥50 events) was specifically encountered in a cluster of 13 tumors corresponding exactly to CCN-HCC (*P* = 1.4 × 10^−11^, Fig. 4b). We validated this association using WGS data from the ICGC-JP series and a subset of 48 samples from the TCGA series (Fig. 4c, Supplementary Data 7). In absence of WGS data for the rest of the TCGA series, we used SNP array data to estimate the number of focal gains (<200 kb) in each tumor as a surrogate marker of the RS1 signature. With a median of 120 events, CCN-HCC displayed a significant increase of focal gains as compared with other HCC in the TCGA series (median = 6, *P* < 2.2 × 10^−16^, Supplementary Fig. 5). Thus, CCN-HCC have a relatively low mutation burden but a large number of structural rearrangements with a specific signature.

### RS1 features suggest a replication stress-induced mechanism

Almost all rearrangements in CCN-HCC belonged to signature RS1, characterized by a combination of small tandem duplications (<100 kb) and inter-chromosomal translocations (Fig. 4d). CCN-HCC also displayed a typical copy-number profile showing hundreds of focal gains, usually one copy above surrounding chromosome segments (Supplementary Fig. 6). Surprisingly, overlaying structural rearrangement breakpoints with copy-number profiles revealed that only 68% of these gains were due to tandem duplications, other gains being frequently surrounded by translocation or inversion breakpoints (Fig. 4e, Supplementary Fig. 6). A recurrent feature consisted of several chromosome segments, usually between 10 and 100 kb, stung together and with the same duplication level relative to their source chromosomes. Most of these events involved segments from two (Fig. 4f) or more (Supplementary Fig. 7) different chromosomes, a feature recently described as templated insertion cycle^23^. Inter-chromosomal templated insertions accounted for 11% of focal gains in CCN-HCC. Other events, which we call intra-chromosomal templated insertions, involved distal segments of a same chromosome and appeared as couples of inversions (Fig. 4f) or duplication and deletion (Supplementary Fig. 7), depending on the orientation of the junctions. Intra-chromosomal templated insertions accounted for 7% of focal gains in CCN-HCC. All these events are consistent with a replication-based mechanism in which a DNA polymerase at a stalled replication fork would switch template, replicate one or more other DNA regions and switch back to the original template strand behind the point of departure, generating a duplication on the host chromosome^23–26^. Such mechanism could be particularly active in CCN-HCC due to replication stress induced by premature S phase entry.

### Structural rearrangements activate *TERT* promoter in CCN-HCC

**Fig. 5:**
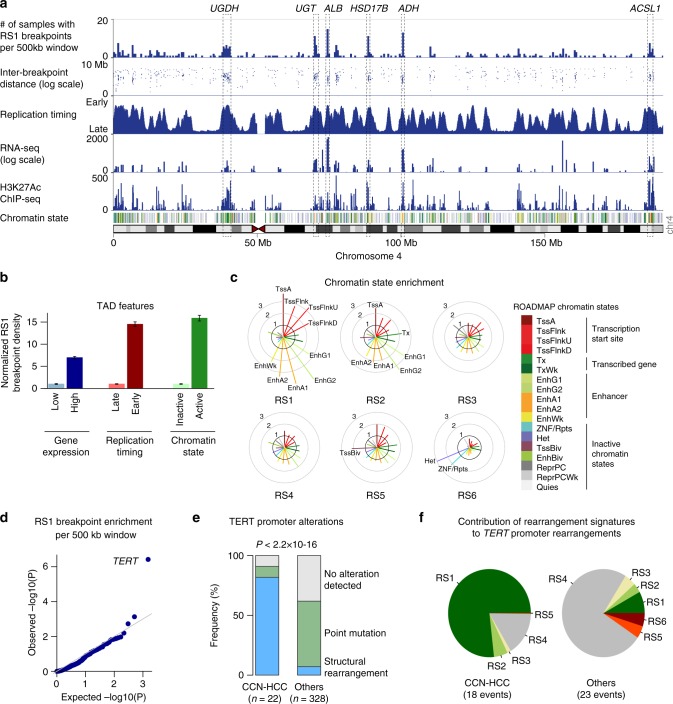
Hotspot analysis of rearrangement signature 1 (RS1) breakpoints. **a** The density of RS1 breakpoints along chromosome 4 is displayed above replication timing, RNA-seq expression, H3K27Ac ChIP-seq profile and chromatin state. Replication timing was determined using Repli-Seq data from the liver cancer cell line HepG2. RNA-seq profile was generated from a normal liver sample. H3K27Ac and chromatin states in normal adult liver were obtained from the ROADMAP consortium. The legend for chromatin state color codes is displayed in **c**. Hotspots corresponding to highly expressed liver enzymes are annotated (UGDH, UDP-glucose 6-dehydrogenase; UGT, UDP glucuronosyltransferase family cluster; ALB, albumin; HSD17B, hydroxysteroid 17-Beta dehydrogenases 11 and 13, ADH, alcohol dehydrogenase cluster; ACSL1, acyl-CoA synthetase long chain family member 1). **b** RS1 breakpoint density in topologically associated domains (TADs). TADs were defined in human embryonic stem cells (H1) and classified based on gene expression in normal liver, replication timing and chromatin state. For each comparison, breakpoint density was normalized to be 1 in the group with the lowest density. Error bars indicate the 95% confidence interval. **c** Enrichment of rearrangement breakpoints in ROADMAP chromatin states for the 6 rearrangement signatures identified in HCC. For each signature, the fold-change between the observed and expected number of breakpoints falling within each chromatin state is represented, and chromatin states with a >2-fold enrichment are annotated. **d** Quantile-quantile plot of RS1 breakpoint enrichment p-values across 500 kb windows. **e** Proportion of *TERT* promoter alterations in CCN-HCC and other HCC analyzed by WGS. **f** Contribution of the 6 rearrangement signatures to *TERT* promoter rearrangements in CCN-HCC and other HCC

To better understand the functional consequences of the rearrangement phenotype observed in CCN-HCC, we examined the location of 8466 breakpoints attributed to the RS1 signature among the 350 liver cancer genomes from the LICA-FR, TCGA and ICGC cohorts. RS1 breakpoints were not distributed evenly along the genome but formed clusters located almost exclusively within active topologically associated domains (TADs, Fig. 5a) characterized by early replication, high gene expression and active chromatin states in normal liver (Fig. 5b). In particular, RS1 breakpoint hotspots were frequently observed at loci encoding very highly expressed liver enzymes exemplified by the albumin (*ALB*), alcohol dehydrogenase (*ADH*) and hydroxysteroid 17-Beta dehydrogenases (*HSD17B*) loci on chromosome 4 (Fig. 5a, Supplementary Fig. 8). Among the 18 chromatin states defined by the ROADMAP consortium in normal adult liver, active transcription start sites (TSS) and enhancer regions were the most strongly enriched in RS1 breakpoints (fold-change > 3), whereas quiescent and heterochromatin domains were the most depleted (Fig. 5c). TSS and enhancer regions were also enriched, to a lesser extent, in breakpoints related to signature RS2 characterized by large tandem duplications. By contrast, breakpoints related to signature RS6, dominated by inversions < 10 kb, were predominantly observed in heterochromatin and ZNF repeats.

We then used binomial regression^27^ to model the density of rearrangement breakpoints along the genome considering an extensive set of genomic features (Supplementary Fig. 9) and to identify hotspots harboring more breakpoints than expected by chance from the background model, which may indicate positive selection in CCN-HCC. We identified a single significant locus corresponding to *TERT* promoter region (*q*=0.0029, Fig. 5d). Although *TERT* promoter mutations were rare in CCN-HCC (9 vs. 55% in others, *P* = 2.4 × 10^−5^), *TERT* promoter rearrangements were highly enriched (82 vs. 7%, *P* = 1.8 × 10^−15^, Fig. 5e) and involved regions of active chromatin in normal liver, in the vicinity of highly expressed liver enzymes (*ALB*, *FGG*, *SEP15*, *SLC12A7* and *BAAT*) or transcription factors (*HNF4A*, *CEBPA*, and *CEBPB*) (Supplementary Data 8, Supplementary Fig. 10). *TERT* promoter rearrangements induced an overexpression of *TERT*, stronger than promoter mutations but lower than HBV insertions (Supplementary Fig. 11). Of the 18 *TERT* promoter rearrangements identified in CCN-HCC, 16 could be associated with signature RS1 with a probability ≥ 0.5 (Fig. 5f). By contrast, most *TERT* promoter rearrangements in other HCC were related to signature RS4. Thus, structural rearrangements induced by replication stress are enriched at active chromatin regions and can promote CCN-HCC development by activating oncogenes like *TERT*.

### CCN-HCC share a similar signature with *BRCA1*-altered cancers

**Fig. 6:**
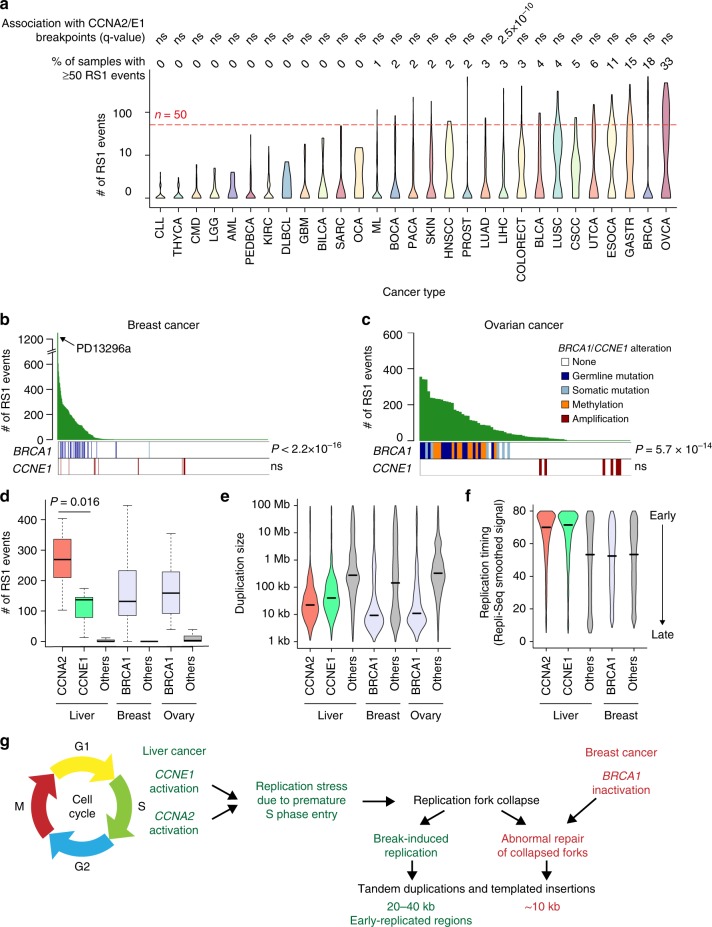
Pan-cancer analysis of the RS1 signature **a** Violin plots representing the number of rearrangements attributed to signature RS1 across patients within each cancer type in the ICGC PCAWG data set. For each cancer type, we assessed the association between tumors with ≥ 50 RS1 events and tumors with a rearrangement breakpoint < 80 kb from *CCNA2* or *CCNE1* gene using Fisher’s exact tests. ns: not significant. The definition of cancer codes and number of samples per cancer type are available in Supplementary Data 9. **b** Number of RS1 events across 524 breast cancer genomes^30^ and association with *BRCA1* alterations and *CCNE1* amplifications. PD13296a, the only tumor with both *BRCA1* mutation and *CCNE1* amplification, has the highest number of RS1 events in the series. **c** Number of RS1 events across 80 ovarian cancer genomes^75^ and association with *BRCA1* alterations and *CCNE1* amplifications. *P*-values were obtained using one-sided Wilcoxon rank-sum tests. **d** Number of RS1 events in liver, breast and ovarian cancers with or without *CCNA2*, *CCNE1* and *BRCA1* alterations. The middle bar, median; box, interquartile range; bars extend to 1.5 times the interquartile range. **e**, Violin plots representing the distribution of tandem duplication sizes across liver, breast and ovarian cancers with or without *CCNA2*, *CCNE1* and *BRCA1* alterations. **f** Violin plots representing the replication timing of duplication and inter-chromosomal translocation breakpoint loci in liver and breast cancers with or without *CCNA2*, *CCNE1* and *BRCA1* alterations. Replication timing was determined using Repli-Seq data from the HepG2 cell line for liver cancer and from the MCF-7 cell line for breast cancer. **g** Proposed connexion beween rearrangement signatures in CCN-HCC and in *BRCA1*-inactivated breast and ovarian cancers

To investigate the prevalence of the RS1 signature in other cancer types, we applied our method to 2606 tumors from the ICGC PanCancer Analysis of Whole Genomes (PCAWG) dataset^23,28,29^. In this pan-cancer series, we identified 9 rearrangement signatures (Supplementary Fig. 12), including one signature (RS1-pancan) highly similar to the RS1 signature that we identified in liver cancers (cosine similarity = 0.91). The RS1-pancan signature was detected at low frequency in several cancer types (e.g. bladder, lung, esophageal and gastric cancers), and was highly active in breast (18% of samples with ≥ 50 RS1 events) and ovarian (33%) cancers. However, this signature was associated with *CCNA2*/*E1* rearrangements only in liver cancer (Fig. 6a, Supplementary Data 9). Thus, the relationship between cyclin A2/E1 activation and signature RS1 is specific to liver cancer, and the molecular cause of this signature in other cancer types remains to be elucidated. In ovarian and breast cancer, RS1 signature was not associated with *CCNE1* amplifications but with *BRCA1* inactivation (Fig. 6b, c), consistent with previous reports^30,31^. Despite sharing a common signature of short tandem duplications and templated insertions, *CCNA2*, *CCNE1* and *BRCA1*-altered tumors displayed slightly different characteristics. First, the number of RS1 rearrangements was higher in *CCNA2*-activated HCC (median = 269) than in *CCNE1*-activated HCC (137) and *BRCA1*-altered breast (132) and ovarian (159) cancers (Fig. 6d). Second, tandem duplications were larger in *CCNE1*-activated HCC (median = 39 kb) than in *CCNA2*-activated HCC (22 kb), and smaller in *BRCA1*-altered breast (9 kb) and ovarian (10 kb) cancers (Fig. 6e). Finally, duplication and translocation breakpoints were strongly enriched in early-replicated regions in CCN-HCC as compared with other HCC, but not in *BRCA1*-altered as compared with other breast and ovarian cancers (Fig. 6f). Cyclin E1 activation was recently shown to induce replication stress by firing novel replication origins located within highly transcribed genes and prone to collapse^32^. BRCA1 is implicated in the response to replication stress^33,34^ and its inactivation leads to tandem duplication formation at stalled forks by a replication restart-bypass mechanism^35^. Cyclin A2/E1 activation in HCC and *BRCA1* inactivation in breast and ovarian cancers may thus converge towards a similar rearrangement signature, with specificities reflecting the different ways by which these genetic alterations induce replication stress or modulate response to it (Fig. 6g).

## Discussion

Here, we report the characterization of a homogeneous HCC subgroup driven by the activation of *CCNA2* or *CCNE1* gene. CCN-HCC represent 7% of HCC across the 3 data sets analyzed here, but up to 14% of HCC developed in a non-fibrotic liver. These patients often have atypical clinical presentation, without any history of primary risk factors, and can be remarkably young, exemplified by tumor FR-3880T developed in a 32 year-old woman without any risk factor, due to a *TET2*-*CCNA2* fusion. CCN-HCC are usually large tumors of poor prognosis but share molecular characteristics, in particular high proliferation and replication stress, that could provide therapeutic opportunities^36^. First, conventional chemotherapies mainly affect actively dividing cells by generating DNA damage or blocking DNA replication, and the tandem duplicator phenotype was identified as a marker for chemotherapeutic response in breast cancer cell lines and patient-derived xenografts^37^. Transarterial chemoembolization (TACE) with doxorubicin, cisplatin or epirubicin, usually recommended for patients with intermediate HCC not eligible for surgery, may thus be an interesting option for CCN-HCC. Poly(ADP-ribose) polymerase (PARP) inhibitors, the first clinically approved drugs designed to exploit synthetic lethality, have demonstrated benefit for patients carrying *BRCA1* mutations^38^. CCN-HCC do not harbor a DNA repair defect but share with *BRCA1*-altered tumors a signature of genomic instability that could conceivably confer these tumors sensitivity to PARP inhibitors. Finally, there are currently several compounds in phase I and II trials targeting the replication stress response pathway members *ATR*, *CHK1* and *WEE1*^39^. If brought to the clinic, such compounds would be promising for CCN-HCC treatment, given that the ATR pathway is strongly upregulated in CCN-HCC and overexpression of *CCNE1* has been shown to confer increased sensitivity to ATR inhibition^40^.

We describe for the first time recurrent fusions involving *CCNA2* gene and recurrent rearrangements of *CCNE1* promoter region. *CCNA2* fusions are only the second recurrent fusion event identified in hepatocellular carcinoma, after the *PRKACA*-*DNAJB1* fusion characteristic of the rare fibrolamellar carcinoma subtype^41^. These fusions always involve the untranslated 5′ region of different partner genes upstream exons 3–8 of *CCNA2*, which constitutes an original mechanism leading to oncogene activation by truncating a regulatory N-terminal domain. Apart from liver cancers, none of the 2606 tumor genomes from the ICGC PCAWG dataset displayed a rearrangement breakpoint in *CCNA2* intron 2. Consistently, a recent RNA-seq analysis of 9,624 TCGA samples from 33 cancer types^42^ did not reveal any *CCNA2* fusion in other cancer types. *CCNA2* fusions thus appear to be specific of liver cancers. Rearrangements affecting *CCNE1* promoter region result in the overexpression of cyclin E1 by bringing active enhancer regions upstream the transcription start site, mirroring the effect of viral enhancers. This mechanism was more frequent than *CCNE1* amplification in the liver cancer series we analyzed. Although HBV and AAV2 insertions were previously identified in *CCNA2* and *CCNE1*^14,16^, the functional consequences of these insertions were unknown. By integrating WGS and RNA-seq data, we demonstrate here that viral insertions in *CCNA2*, like gene fusions, induce abnormal transcripts leading to truncated proteins lacking N-terminal regulatory domains. By contrast, viral insertions in *CCNE1* region lead to the overexpression of a full-length transcript and protein.

CCN-HCC display a characteristic transcriptional program, with a strong overexpression of E2F targets. Activation of the E2F pathway is expected in *RB1*-altered tumors and was already described in HCC^43^. However, E2F pathway is also activated in CCN-HCC without *RB1* inactivation event and may be partly explained by the ability of cyclin E/Cdk2 complexes to phosphorylate Rb. Interestingly, E2F-1 overexpression in the liver causes dysplasia and tumors in mice^43^, and E2F1 was shown to inhibit c-Myc-driven apoptosis by activating PIK3CA/Akt/mTOR and c-Myb/COX-2 pathways^44^.

A striking feature of CCN-HCC is the accumulation of hundreds of tandem duplications and templated insertion cycles. A recent study showed that *CCNE1* activation in U2OS cell lines leads to shortened G1 phase, early S phase entry and firing of normally silenced replication origins in highly expressed genes, prone to collapse and associated with DNA double-strand breaks formation^32^. Double-strand breaks formed following replication fork breakdown are primarily repaired by break-induced replication (BIR)^45^. In a cyclin E overexpression model of DNA replication stress, BIR was shown to be required for cell cycle progression and to induce duplications < 200 kb^46^. In addition, template switching may occur during BIR and generate complex chromosome rearrangements^24,25,47^. Thus, the nature of rearrangements identified in CCN-HCC and the enrichment of breakpoints in early-replicated, actively transcribed regions are consistent with a BIR mechanism induced by replication stress. However, future studies addressing the precise molecular mechanism generating templated insertions will be crucial to fully understand the relationship between replication stress and the RS1 rearrangement signature. The mechanism of tandem duplication formation in *BRCA1*-mutant cells was recently identified^35^. It involves abnormal repair of collapsed replication forks by a “replication restart bypass” mechanism with extension of the stalled leading strand by a migration bubble mechanism similar to BIR^48^, terminated by end joining or by microhomology-mediated template switching. Thus, structural rearrangements induced by cyclin activation and *BRCA1* deficiency are initiated by replication fork collapse and processed by different repair mechanisms leading to a similar rearrangement signature with subtle differences regarding the size of rearrangements and breakpoint location. Interestingly, *BRCA1* inactivation and *CCNE1* amplification are mutually exclusive in ovarian cancers^49^, and have been shown to be synthetically lethal^50^. The single breast tumor that we identified with both *BRCA1* mutation and *CCNE1* amplification (PD13296a) had the highest number of rearrangements related to the RS1 signature (*n* = 1221) across all the tumors we analyzed.

Contrary to *CCNA2* alterations that seem to be specific of liver cancers, *CCNE1* activation by high-level amplification is frequent across human cancers, in particular in gynecologic cancers^51^. Yet, *CCNE1* amplification in breast and ovarian cancers does not lead to the rearrangement phenotype that we observed in CCN-HCC. Several reasons may explain this discrepancy. First, adult hepatocytes are quiescent, rarely divide, and may thus be particularly sensitive to replication stress. Second, *CCNE1* is mostly activated by viral insertions and structural rearrangements of regulatory regions in HCC, rather than chromosome amplifications. These alterations may not have exactly the same functional consequence. Finally, we believe that viral insertions and structural rearrangements activating *CCNA2* or *CCNE1* are early events triggering hepatocarcinogenesis because they occur in patients without cirrhosis and in absence of other oncogenic event like *CTNNB1* mutations. *CCNE1* amplifications may occur later in breast and ovarian tumors, not leaving enough time for rearrangements to accumulate. Fujimoto et al. reported a positive correlation between the number of structural rearrangements and HBV insertion sites, suggesting that double-strand breaks generated by structural rearrangements may provide opportunities for HBV integration^11^. Here we describe the reciprocal relationship where viral insertions in cyclin genes lead to structural rearrangement formation due to replication stress.

The propensity of signature RS1 breakpoints to occur in enhancer-rich regions makes these rearrangements likely to activate oncogenes in trans. In this limited series of 22 CCN-HCC analyzed by WGS, we identified a single significantly recurrent hotspot at *TERT* promoter. However, the power to identify recurrent somatic rearrangement hotspots increases sharply with sample size^27^, and future studies of larger CCN-HCC series may uncover additional sites under positive selection in CCN-HCC.

In conclusion, viral insertions and structural rearrangements activating *CCNA2* and *CCNE1* define a homogeneous subgroup of aggressive HCC developed in non-cirrhotic liver, sharing similar transcriptional profiles and frequent inactivation of *RB1* and *PTEN*. These tumors display a specific rearrangement signature induced by replication stress that sustains tumor growth by activating *TERT* but may constitute a targetable vulnerability.

## Methods

### Description of the LICA-FR cohort

A series of 160 hepatocellular carcinoma (HCC) samples and their non-tumor counterparts were collected from patients surgically treated in four French hospitals located in Bordeaux and Paris region. The study was approved by institutional review board committees (CCPRB Paris Saint-Louis, 1997, 2004, and 2010, approval number 01–037; Bordeaux, 2010-A00498–31). Written informed consent was obtained in accordance with French legislation. All samples were immediately frozen in liquid nitrogen and stored at −80 °C. HCC were enriched in cases developed on a non-cirrhotic liver (107/160, 67%): 75 tumors developed in non-fibrotic (METAVIR F0-F1), 32 in chronic hepatitis (F2–F3) and 53 in cirrhotic liver (F4). Clinicopathological data were available for all cases. A diversity of risk factors were represented in our series, including alcohol (*n* = 63), metabolic syndrome (*n* = 37), HBV (*n* = 30), and HCV infection (*n* = 30). Twenty-nine patients had none of the above risk factors. These 160 samples were analyzed by RNA sequencing, 156 were analyzed by whole exome sequencing (including 96 were previously published^10^) and 45 by whole genome sequencing (35 were previously published^22^). Detailed clinical characteristics and sequencing details for each sample are provided in Supplementary Data 1.

### Whole genome sequencing

Whole genome data from 45 tumors of the LICA-FR series were analyzed in this study, comprising 35 previously published^22^ and 10 new cases. The whole genomes of 10 new tumor/normal pairs were sequenced for this project at the Center National de Recherche en Génomique Humaine (CNRGH, Evry, France) on an Illumina HiSeq X Five as paired-end 151 bp reads. Sequences were aligned to the hg19 version of the human genome using BWA^52^ version 0.7.12. We used Picard tools version 1.108 (http://broadinstitute.github.io/picard/) to remove PCR duplicates and GATK^53^ version v3.5 for local indel realignment and base quality recalibration, as recommended in GATK best practices^54^. We obtained an average depth of 119-fold for tumors (range 104–126) and 41-fold for matched non-tumor liver samples (range 38–43).

### Whole exome sequencing

Whole exome data from 156 tumors of the LICA-FR series were analyzed in this study, comprising 96 previously published^10^ and 60 new cases. Sequence capture, enrichment and elution of genomic DNA samples from the 60 new tumor/normal pairs was performed by IntegraGen (Evry, France). Agilent in-solution enrichment was used with the manufacturer’s biotinylated oligonucleotide probe library SureSelect Human All-Exon kit v5 + UTRs (*n* = 39) or SureSelect Clinical Research Exome V2 (*n* = 21) according to the manufacturer’s instructions. The eluted enriched DNA sample was sequenced on an Illumina HiSeq 2000 (*n* = 39) or HiSeq 4000 (*n* = 21) as paired-end 75 bp reads. Sequencing details for each sample are indicated in Supplementary Data 1.

### Somatic mutation calling

We used MuTect2 to call somatic mutations from WES and WGS data by comparing each tumor sample with its matched non-tumor counterpart and a panel of normals (PON) file. We excluded mutations belonging to the ENCODE Data Analysis Consortium blacklisted regions (http://hgdownload.cse.ucsc.edu/goldenPath/hg19/encodeDCC/wgEncodeMapability/wgEncodeDacMapabilityConsensusExcludable.bed.gz) and regions covered by < 6 reads in the tumor or normal sample. We then selected only single nucleotide variants (SNVs) with a MuTect2 flag among “PASS”, “clustered_events”, “t_lod_fstar”, “alt_allele_in_normal” or “homologous_mapping_event” and small insertions and deletions (indels) with a MuTect2 flag among “PASS”, “clustered_events” or “str_contraction”. To improve specificity in the calling of mutations with low variant allele frequency (VAF), we quantified the number of high quality variant reads in the tumor (mapping quality ≥ 20, base quality ≥ 20) and the number of variant reads in the non-tumor sample with no quality threshold using bamreadcount (https://github.com/genome/bam-readcount). Only variants matching the following criteria were finally retained: VAF ≥ 2% in the tumor with ≥ 3 variant reads, VAF ≤ 5% in the non-tumor samples with ≤ 2 variant reads, and a VAF ratio ≥ 5 between the tumor and non-tumor sample.

### Copy-number and structural rearrangement analysis

We used MANTA^55^ software to identify somatic structural rearrangements in WGS data. To keep only the most reliable events, we selected only rearrangements supported by ≥ 10 reads and with a variant allele fraction ≥ 5%. We used cgpBattenberg^56^ algorithm to reconstruct copy-number profiles from WGS data. We used the circular binary segmentation algorithm implemented in the Bioconductor package DNAcopy^57^ to reconstruct copy-number profiles from WES data.

### RNA sequencing

RNA samples from the 160 tumors of the LICA-FR series were sequenced in several batches with slightly different protocols. RNA samples were enriched for polyadenylated RNA from 5 μg of total RNA, and the enriched samples were used to generate sequencing libraries with the Illumina TruSeq or Illumina TruSeq Stranded mRNA kit and associated protocol as provided by the manufacturer. Libraries were sequenced by IntegraGen (Evry, France) on an Illumina HiSeq 2000 or 4000 as paired-end 75 or 100 bp reads. Full Fastq files were aligned to the reference human genome hg19 using TopHat2^58^. Sequencing details for each sample and the parameters used for TopHat2 are indicated in Supplementary Data 1. We removed reads mapping to multiple locations, and we used HTSeq^59^ to obtain the number of reads associated to each gene in the Gencode v19 database, restricting to protein-coding genes, pseudogenes, antisense and lincRNAs (*n* = 42540). We used the Bioconductor DESeq2 package^60^ to import raw HTSeq counts for each sample into R statistical software and apply variance stabilizing transformation (VST) to the raw count matrix. FPKM scores (number of fragments per kilobase of exon model and millions of mapped reads) were calculated by normalizing the count matrix for the library size and the coding length of each gene. We used the area under the ROC curve (AUC) to identify and remove 2724 genes with a significant batch effect (AUC > 0.95 between one sequencing project and others).

### Gene fusion detection

Fusions detected by TopHat2 (--fusion-search --fusion-min-dist 2000 --fusion-anchor-length 13 --fusion-ignore-chromosomes chrM) were filtered using the TopHatFusion-post algorithm. We kept only fusions validated by BLAST and with at least 10 split-reads or pairs of reads spanning the fusion event, and we removed fusions identified at least twice in a cohort of 36 normal liver samples.

### Gene expression analysis

We used t-distributed stochastic neighbor embedding (t-SNE) to classify HCC based on their gene expression profiles. We selected the 1000 most variably expressed genes, and we used 1 minus the weighted Pearson correlation coefficient as the distance measure. Pairwise Pearson correlation was calculated using the wtd.cors function of the *weights* R package. We used standard deviation substracted by 0.2 as the weight, giving more variable genes greater influence. The resulting distance matrix was used to perform the t-SNE analysis using the R package *Rtsne*^61^ with default parameters except the following: theta = 0, is_distance = T, pca = F, max_iter = 2000. We used the Bioconductor *limma* package^62^ to test for differential expression between CCN-HCC and other HCC of all genes expressed in at least five samples (FPKM > 0). We applied a *q*-value threshold of ≤ 0.05 to define differentially expressed genes. We used an in-house adaptation of the GSEA method^63^ to identify gene sets from the MSigDB v6 database overrepresented among upregulated and downregulated genes.

### Viral insertion screening

AAV2 insertions had previously been screened by viral capture and whole exome sequencing in 83 tumors from the LICA-FR cohort^16^. We extended this screen to AAV2 and HBV insertions in all HCC from the LICA-FR cohort using RNA-seq and WES data. In the ICGC-JP cohort, AAV2 and HBV insertions had already been screened using WGS data and were provided by Fujimoto et al.^11^ In the TCGA cohort, we screened AAV2 and HBV insertions using RNA-seq data from all tumors and WES data from 37 tumors showing viral reads or overexpression of *CCNA2* or *CCNE1* in RNA-seq data. For each tumor and matched normal sample, the sequence reads were mapped to the AAV2 (AF043303.1) and HBV (X02763, renumbered using the EcoR1 restriction site as the +1) reference genomes using BWA^52^. Read pairs with at least one read aligned on the virus were extracted using samtools^64^, and aligned to a custom reference genome including human chromosomes and virus fasta sequences as pseudo-chromosomes. Tumors with ≥ 6 chimeric reads or read pairs aligned on both the human and viral genomes were further analyzed. All viral insertions were validated by visual inspection on IGV^65^. We used chimeric reads to identify insertion breakpoints at base resolution by mapping sequences on both sides of the junctions. Of the 12 LICA-FR tumors with viral insertions detected in *CCNA2* or *CCNE1*, 7 were previously analyzed by viral capture sequencing^16^ and 3 were analyzed by whole genome sequencing. For these 10 tumors, we were able to extract reads covering the full length of the inserted viral genome and to reconstruct the complete human-virus-human chimeric sequence.

### Consequences of cyclin A2 alterations on protein structure

All tumors from the LICA-FR series harboring AAV2 or HBV insertions in *CCNA2* were analyzed by WGS or viral capture^16^ to determine the precise boundaries of viral insertion breakpoints. RNA-seq reads were then aligned on the reconstructed chimeric sequence with TopHat2^58^, and we used Cufflinks v2.2.1^66^ to identify and quantify the different transcripts. We used ElemeNT^67^ to predict transcription initiation sites and Alamut Visual software (Interactive Biosoftware) to identify splicing signals on the chimeric DNA sequence. We used ATGpr^68^ to identify translation initiation sites on abnormal transcripts resulting from viral insertion or gene fusions.

### Western blot analysis of cyclin A2 and cyclin E1 proteins

Cell protein extracts were prepared using hot Laemmli buffer (50 mM Tris, pH = 6.8, 2% SDS, 5% glycerol, 2 mM DTT, 2.5 mM EDTA, 2.5 mM EGTA, Protease inhibitor cocktail complete MINI EDTA-free (Roche Applied Science), 1× HALT Phosphatase inhibitor (Perbio), 2 mM Na3VO4 and 10 mM NaF). Protein concentration was assessed using the BCA Protein Assay Kit (Pierce). Western blot analyses were conducted using the following primary antibodies: CCNA2 N-ter (#211735, Abcam); CCNA2 C-ter (#32386, Abcam), CCNE1 (#33911, Abcam), and β-actin (#4967, Cell Signaling Technology) used as loading control. Proteins of interest were detected using an anti-rabbit IgG horseradish peroxidase–linked secondary antibody (#7074, Cell Signaling Technology) and the ECL Chemiluminescence Western Blotting Detection Kit (GE Healthcare), according to the provided protocol. Signal detection was performed using the ChemiDoc XRS system and the Image Lab software (Bio-Rad). All antibodies were used at 1:1000 dilution except secondary antibody, which was used at 1:2000.

### Mutational and rearrangement signature analysis

We used the *Palimpsest* R package^69^ to extract mutational and rearrangement signatures from WGS data. For point mutations, we quantified the contribution of the 10 mutational signatures referenced on the COSMIC website (https://cancer.sanger.ac.uk/cosmic/signatures) and described as operative in liver cancers (signatures 1, 4, 5, 6, 12, 16, 17, 22, 23, 24)^22^ to each tumor genome. For structural rearrangements, we performed a de novo signature analysis across the 350 HCC genomes from the LICA-FR, TCGA and ICGC-JP datasets. We identified 6 rearrangement signatures that were very similar to the 6 signatures we previously obtained on a smaller dataset^22^, except that the two initially described deletion signatures were now merged into signature RS5, and that a new signature emerged (RS6, dominated by inversions < 10 kb). We used *Palimpsest* to quantify the contribution of each signature to each tumor genome and to estimate the probability of each structural rearrangement being due to each process.

### Identification of rearrangement hotspots

We identified 8466 breakpoints attributed to signature RS1 (probability > 0.5) across the 350 HCC genomes from the LICA-FR, TCGA and ICGC-JP datasets. To account for the uneven distribution of rearrangements in the genome, we then modeled the background distribution of breakpoints considering various genomic features as described by Glodzik et al.^27^, with some modifications. In short, we divided the genome into 500 kb bins, and we characterized for each bin 17 genomic features likely to influence the density of rearrangements: replication timing in HepG2 cell line (ENCODE^70^), highly expressed (top 25%) and low-expressed (remaining 75%) genes in normal liver, average copy-number in the cohort, repetitive sequences (segmental duplications, ALU elements and other repeats), number of N bases in the reference genome, known fragile sites^71^, chromatin staining, DNAse hyper-sensitive sites and 6 histone marks (H3K4me1, H3K4me3, H3K9me3, H3K27me3, H3K36me3, H3K27ac) in adult liver (ROADMAP^72^). All features were normalized to a mean of 0 and standard deviation of 1 across the bins. The total number of RS1 breakpoints were counted for each bin, and we used negative binomial regression to model the distribution of breakpoints according to the 17 normalized features. The model was trained across 4993 bins after removing bins containing validated cancer genes from the Cancer Gene Census^73^ (https://cancer.sanger.ac.uk/census). For signature RS1, the most predictive features of a high breakpoint density were DNAse accessibility, H3K27 acetylation and early replication timing. We then used this model to estimate the expected number of breakpoints across 761 bins containing cancer genes, and we compared the number of observed breakpoints to the number of expected breakpoints using a one-sided binomial test. Finally, p-values were corrected for multiple testing using Benjamini-Hochberg procedure.

### Chromatin state analysis

We used various genomic features to correlate with structural rearrangement density and to better understand the functional consequences of rearrangements. We used replication sequencing (Repli-seq) wavelet-smoothed signals downloaded generated by the ENCODE^70^ consortium for the liver cancer cell line HepG2 to define early and late-replicating regions. We used ChIP-seq data for various histone modifications (H3K4me1, H3K4me3, H3K9me3, H3K27me3, H3K36me3, H3K27ac) and chromatin states derived from these modifications in normal adult liver by the ROADMAP consortium^72^. Topologically associated domain (TAD) boundaries in human embryonic stem cells (H1) were provided by Tsirigos et al.^74^

### Pan-cancer analysis of structural rearrangement signatures

Somatic structural rearrangements called by a uniform pipeline over 2,606 tumor genomes were downloaded from the ICGC PanCancer Analysis of Whole Genomes (PCAWG) project^23,28,29^. Using *Palimpsest*^69^, we identified 9 rearrangement signatures in this data set, including one (RS1-pancan) very similar to the RS1 signature identified in CCN-HCC, and we quantified the contribution of each signature to each tumor genome. In each cancer type, we tested if the presence of ≥ 50 rearrangements attributed to signature RS1-pancan was associated with the presence of rearrangement breakpoints < 80 kb from *CCNA2* or *CCNE1* gene using Fisher’s exact test. We analyzed two additional series of breast (*n* = 524)^30^ and ovarian (*n* = 80)^75^ cancer genomes to correlate the amount of RS1-pancan events with *CCNE1* amplifications and *BRCA1* alterations.

### Clinical associations

We tested the association of CCN-HCC in the LICA-FR cohort with gender, age, etiology, liver fibrosis, Edmonson grade, and vascular invasion using Wilcoxon rank sum test for continuous variables, Fisher’s exact test for binary variables and Chi square test for trend for categorical variables. We used log-rank test and Kaplan–Meier method to compare overall survival between CCN-HCC and others, considering only HCC with curative resection (R0) and excluding patients who died within 3 months after surgery.

### Computing codes

The functions used to perform the signatures analysis and associated figures are available as an open-source R package, Palimpsest, available on Github: https://github.com/FunGeST/Palimpsest.

### URLs

ICGC data portal, https://dcc.icgc.org/; COSMIC database, https://cancer.sanger.ac.uk/cosmic; ENCODE project, https://www.encodeproject.org; GENCODE v19, http://www.gencodegenes.org/releases/19.html; ROADMAP project, http://www.roadmapepigenomics.org; NCI GDC data portal, https://portal.gdc.cancer.gov.

## Electronic supplementary material

Supplementary Information

Description of Additional Supplementary Files

Supplementary Data 1

Supplementary Data 2

Supplementary Data 3

Supplementary Data 4

Supplementary Data 5

Supplementary Data 6

Supplementary Data 7

Supplementary Data 8

Supplementary Data 9

Reporting Summary

## Data Availability

The sequencing data reported in this paper have been deposited to the EGA (European Genome-phenome Archive) database (RNA-seq accession [EGAS00001002879]; WES accessions [EGAS00001000217], [EGAS00001001002] and [EGAS00001003063]; WGS accessions [EGAS00001002408], [EGAS00001000706] and [EGAS00001002888]) and the Inter- national Cancer Genome Consortium (ICGC) data portal (http://dcc.icgc.org/; release 27, April 2018).
