# Supplementary material for: Cyclin A2/E1 activation defines a hepatocellular carcinoma subclass with a rearrangement signature of replication stress
Source: Nat Commun. 2018 Dec 7;9:5235. doi: 10.1038/s41467-018-07552-9 (PMC6286353; doi:10.1038/s41467-018-07552-9)
Supplement: Supplementary file 1 — Supplementary Information [file 41467_2018_7552_MOESM1_ESM.pdf]

Supplementary Information

Cyclin A2 and E1 genomic alterations define a specific subclass of hepatocellular carcinomas

Bayard *et al.*

Supplementary Figures

Supplementary Figure 1. Consequences of viral insertions in *CCNA2* gene
Supplementary Figure 2. Deletions associated with *CCNA2* deregulation
Supplementary Figure 3. Cyclin E1 overexpression induced by viral insertions and structural rearrangements
Supplementary Figure 4. Mutational signature analysis of CCN-HCC
Supplementary Figure 5. SNP array analysis of focal duplications in CCN-HCC from the TCGA series
Supplementary Figure 6. Characteristic copy-number profile of CCN-HCC
Supplementary Figure 7. Examples of intra-chromosomal templated insertions and templated insertion cycles involving several chromosomes
Supplementary Figure 8. RS1 breakpoint hotspots involving highly expressed liver enzymes
Supplementary Figure 9. Binomial regression modeling of rearrangement breakpoint density
Supplementary Figure 10. Rearrangements affecting *TERT* promoter region in CCN-HCC
Supplementary Figure 11. Modulation of *TERT* expression by different types of genomic alterations.
Supplementary Figure 12. Rearrangement signatures identified in the pan-cancer series

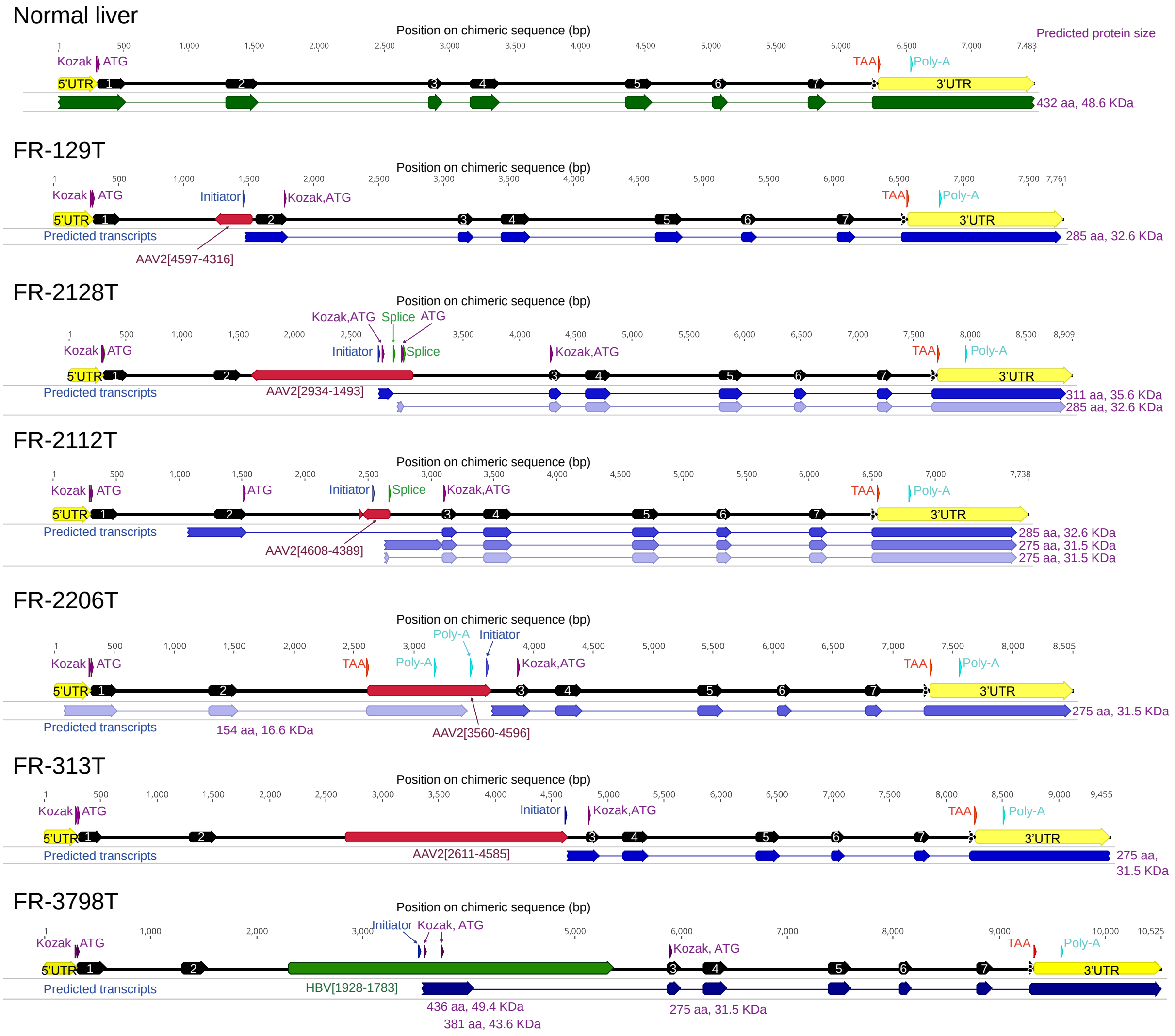

Supplementary Figure 1. Consequences of viral insertions in *CCNA2* gene

Five AAV2 and 1 HBV insertions were identified in *CCNA2* in the LICA-FR series. Precise insertion boundaries were identified by WGS or viral capture, and RNA-seq reads were aligned on the chimeric sequence. Here, the different transcripts predicted by Cufflinks are represented for each case, ordered by transcript abundance. Predicted functional elements (Transcription initiator, splice and poly-A sites, Kozak sequence, initiator and terminator codons) are annotated on the chimeric DNA sequence, and the predicted protein sizes resulting from the translation of each transcript are annotated on the right. Only the most abundant abnormal transcripts were represented in **Fig. 1c**.

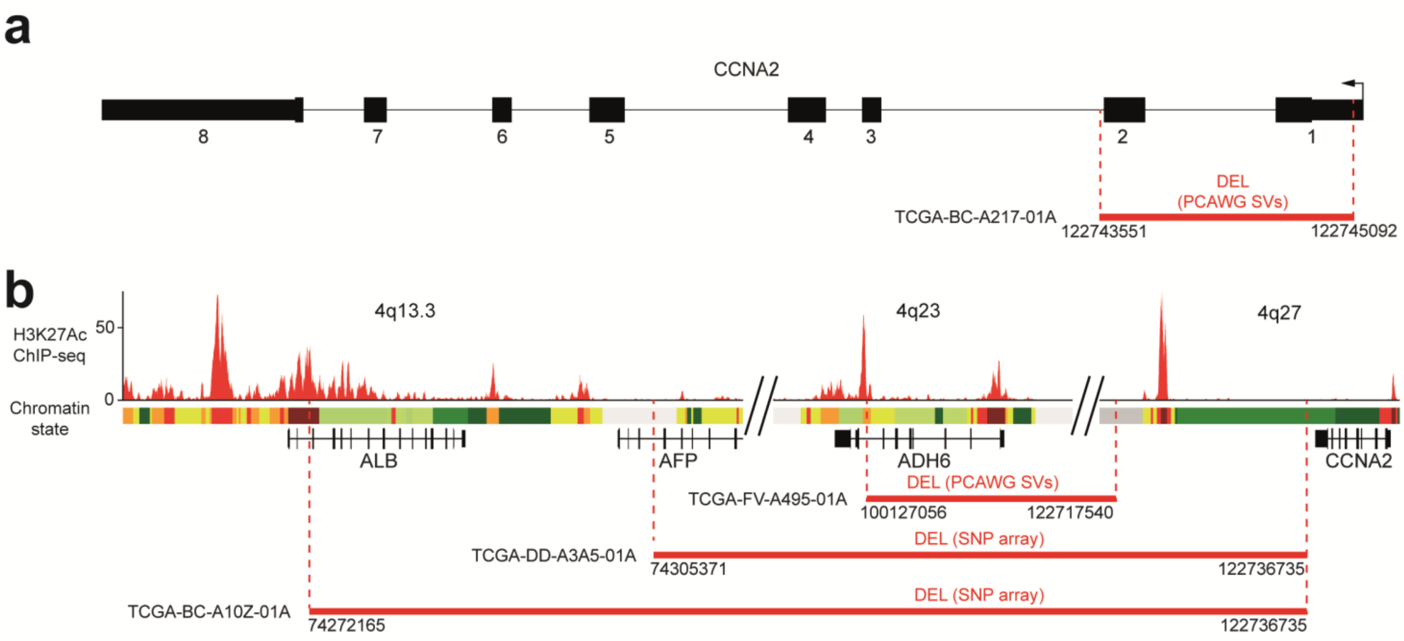

Supplementary Figure 2. Deletions associated with *CCNA2* deregulation
a Focal deletion of *CCNA2* exons 1 & 2 identified in TCGA tumor TCGA-BC-A217.
b Deletions identified in 3 TCGA tumors linking *CCNA2* downstream region with the highly expressed genes *ALB*, *AFP* and *ADH6*.

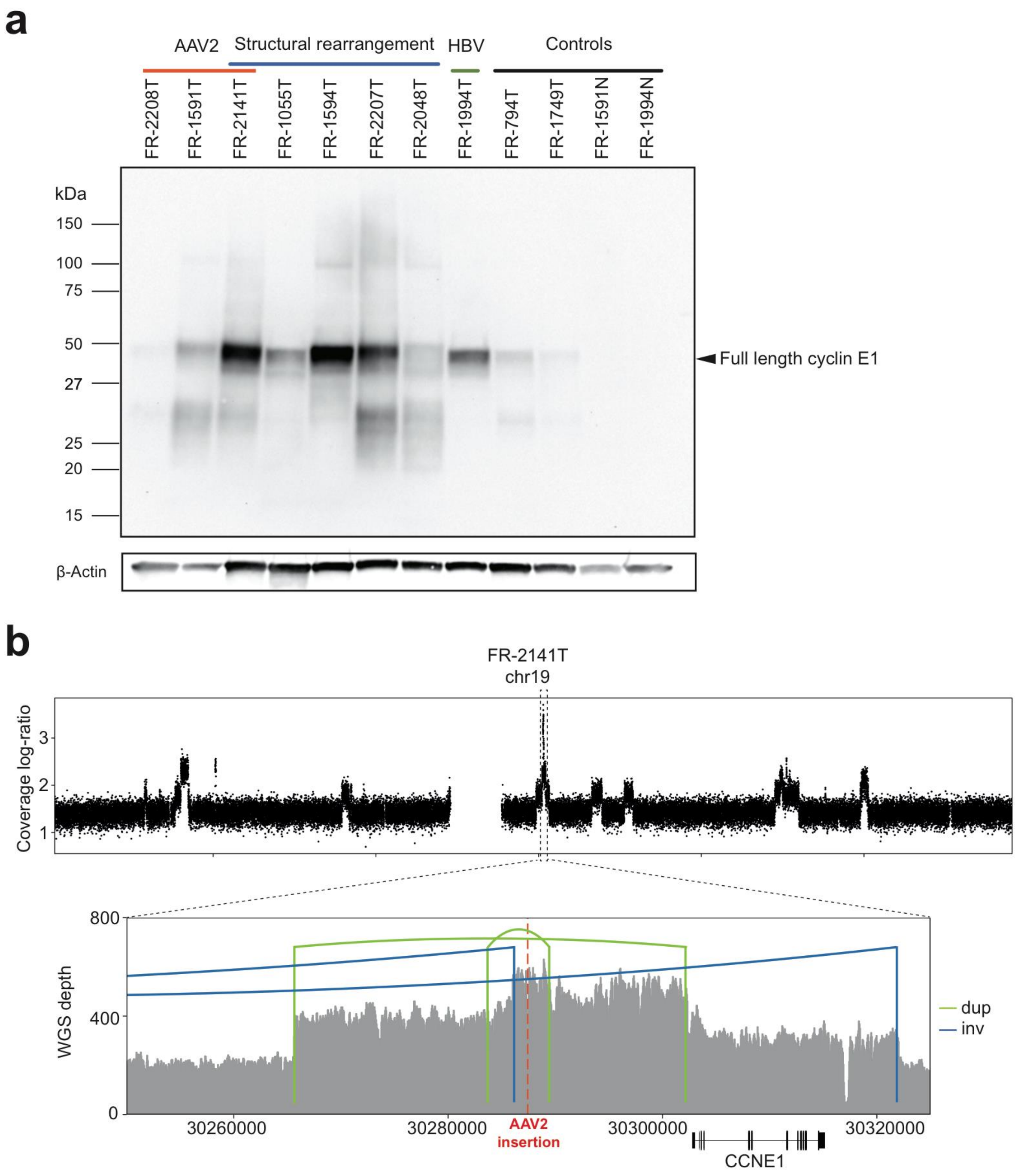

Supplementary Figure 3. Cyclin E1 overexpression induced by viral insertions and structural rearrangements

a Western blot analysis of cyclin E1. Tumors with viral insertions or structural rearrangements are compared with tumors without *CCNE1* alteration and non-tumoral liver controls.

b Tumor FR-2141T displays both an AAV2 insertion in *CCNE1* regulatory region and a high-level amplification of the locus. The top panel displays the coverage log-ratio along chromosome 19 in this tumor. The bottom panel displays the coverage of WGS reads aligned to the chimeric sequence of *CCNE1* locus including AAV2 insertion together with structural rearrangement breakpoints. It shows that the most strongly amplified region includes *CCNE1* regulatory region, and in particular the locus of AAV2 insertion.

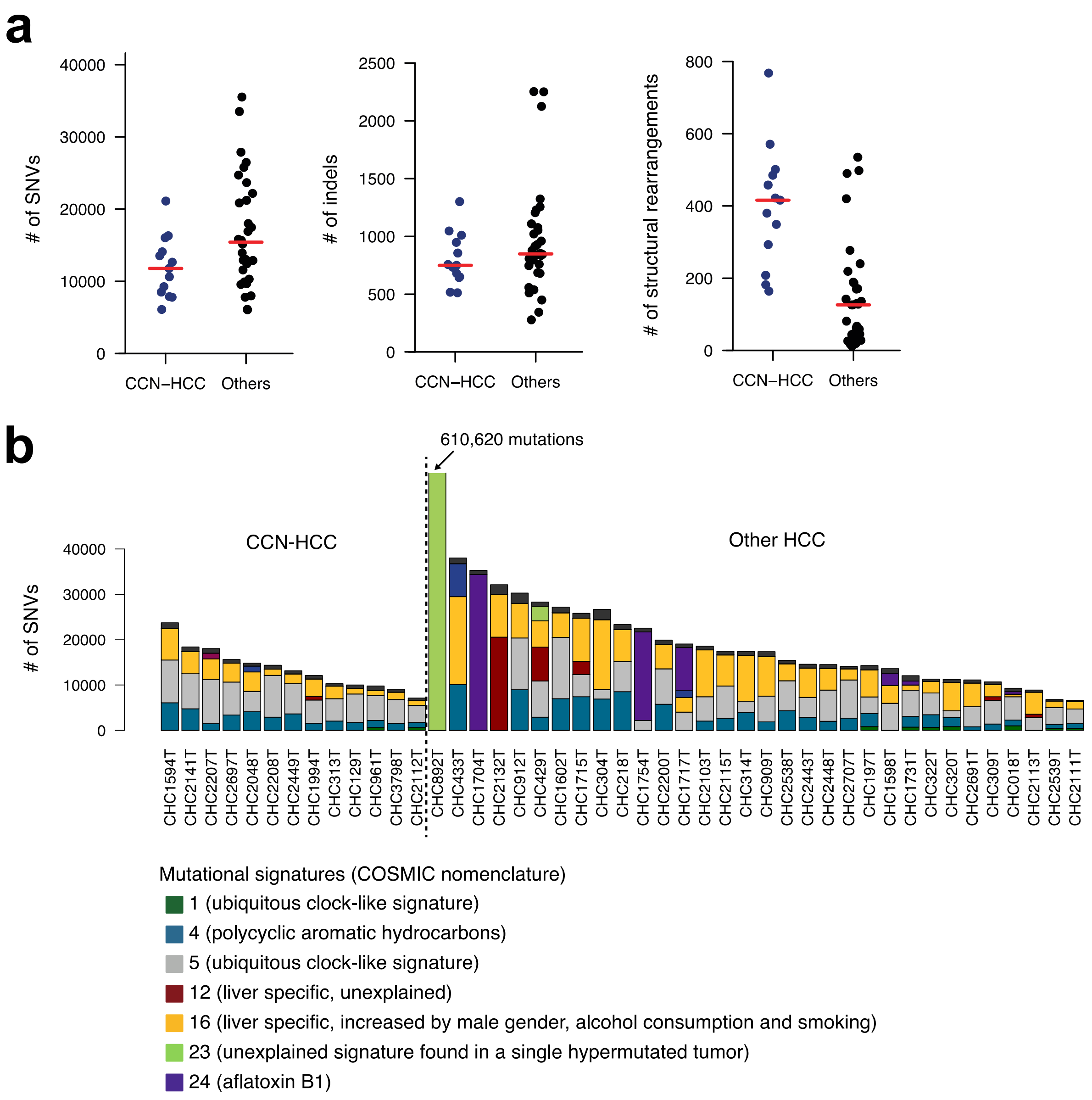

Supplementary Figure 4. Mutational signature analysis of CCN-HCC

a Comparison of the number of single-nucleotide variants (SNVs), indels and structural rearrangements in CCN-HCC vs others (LICA-FR data, 45 WGS). Red line represent median.

b Contribution of the different mutational signatures known to be operative in liver cancers to the mutational burden of CCN-HCC and other HCC.

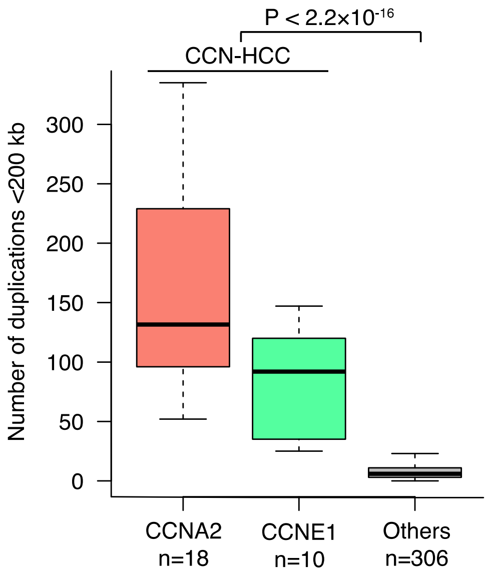

Supplementary Figure 5. SNP array analysis of focal duplications in CCN-HCC from the TCGA series.

Focal deletions (<200 kb) were quantified across 334 tumors from the TCGA series as a surrogate marker of the RS1 signature, and compared between CCNA2-activated, CCNE1-activated and other HCC. The middle bar, median; box, interquartile range; bars extend to 1.5 times the interquartile range.

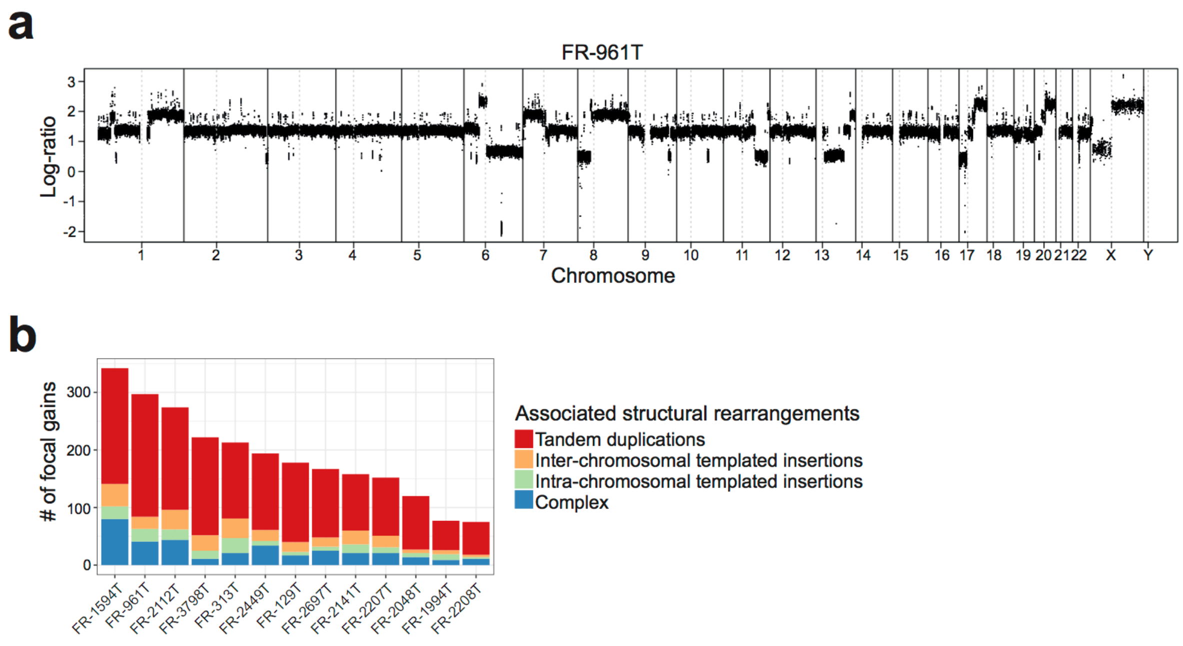

Supplementary Figure 6. Characteristic copy-number profile of CCN-HCC

a Typical copy-number profile of a CCN-HCC, showing hundreds of focal gains scattered throughout the genome.

b Proportion of focal gains in each CCN-HCC attributed to each rearrangement mechanism.

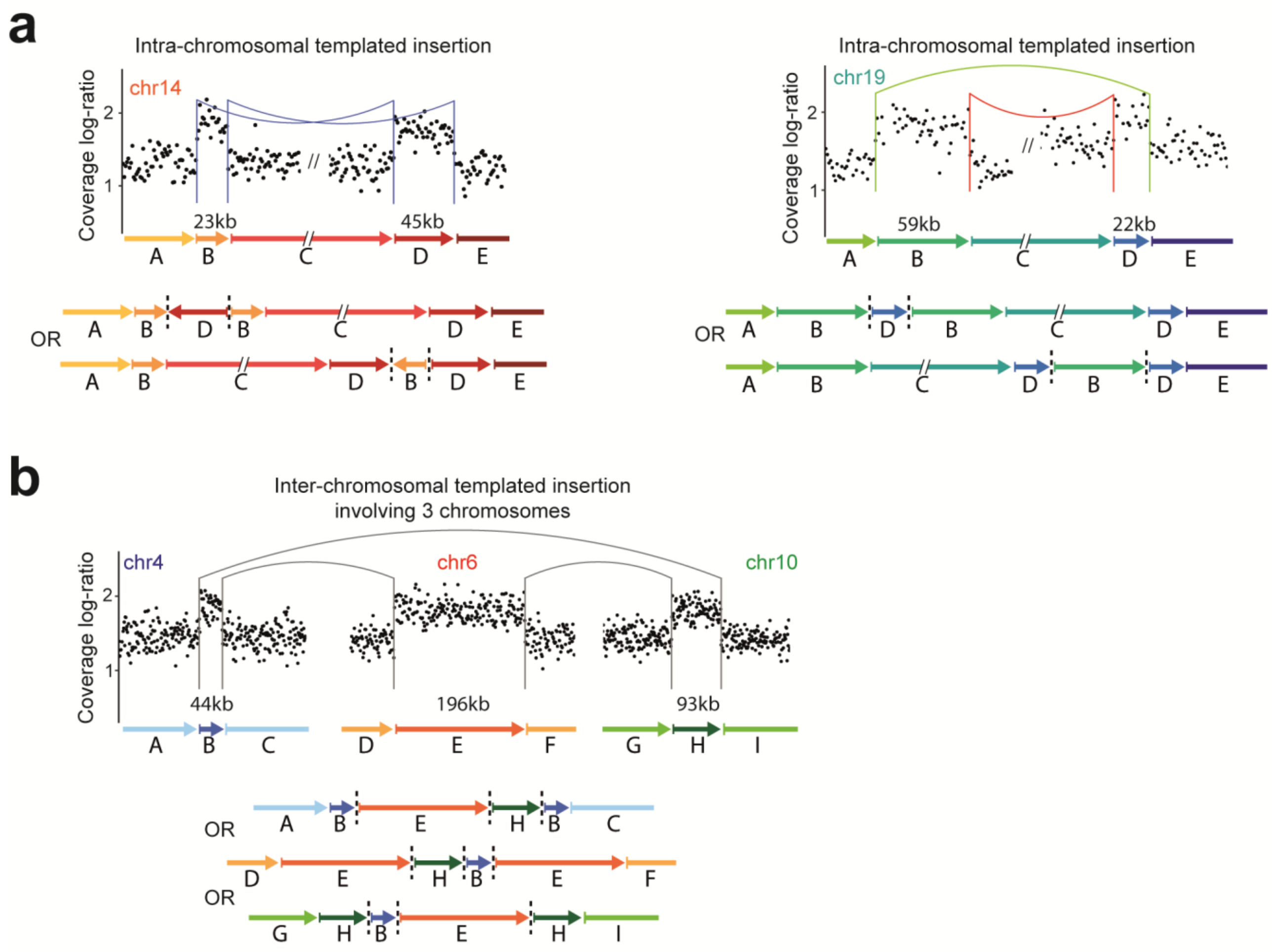

Supplementary Figure 7. Examples of intra-chromosomal templated insertions and inter-chromosomal templated insertion cycles involving several chromosomes

a Intra-chromosomal templated insertions appear as couples of inversions (left) or deletion and duplication (right) depending on the orientation of aberrant junctions.

b Example of inter-chromosomal templated insertion involving 3 different chromosomes.

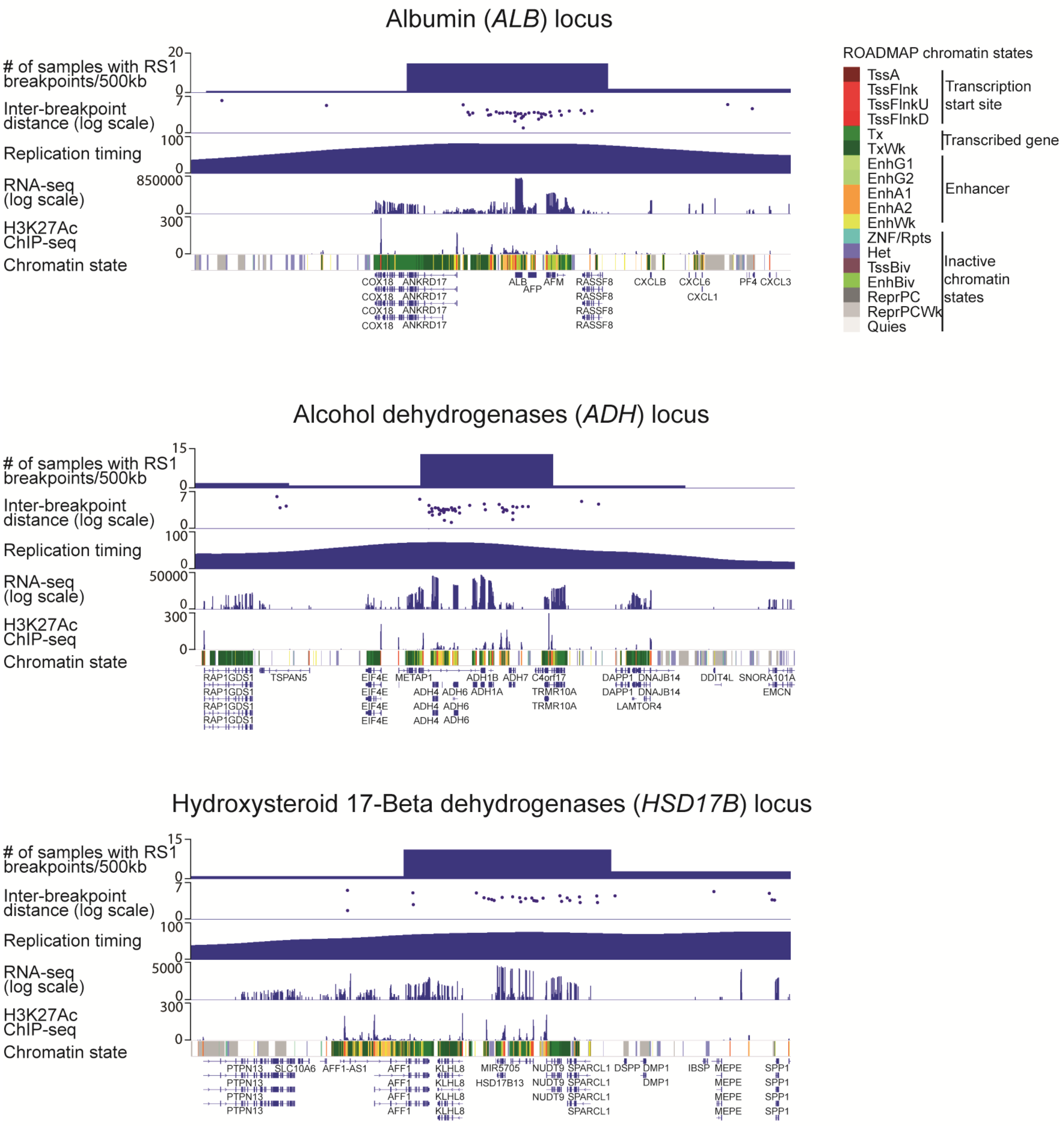

Supplementary Figure 8. RS1 breakpoint hotspots involving highly expressed liver enzymes

The density of RS1 breakpoints, replication timing, RNA-seq expression, H3K27Ac and ROADMAP chromatin states are displayed for 3 representative hotspots involving the very highly expressed liver enzymes albumin, alcohol dehydrogenases and hydroxysteroid 17-Beta dehydrogenases.

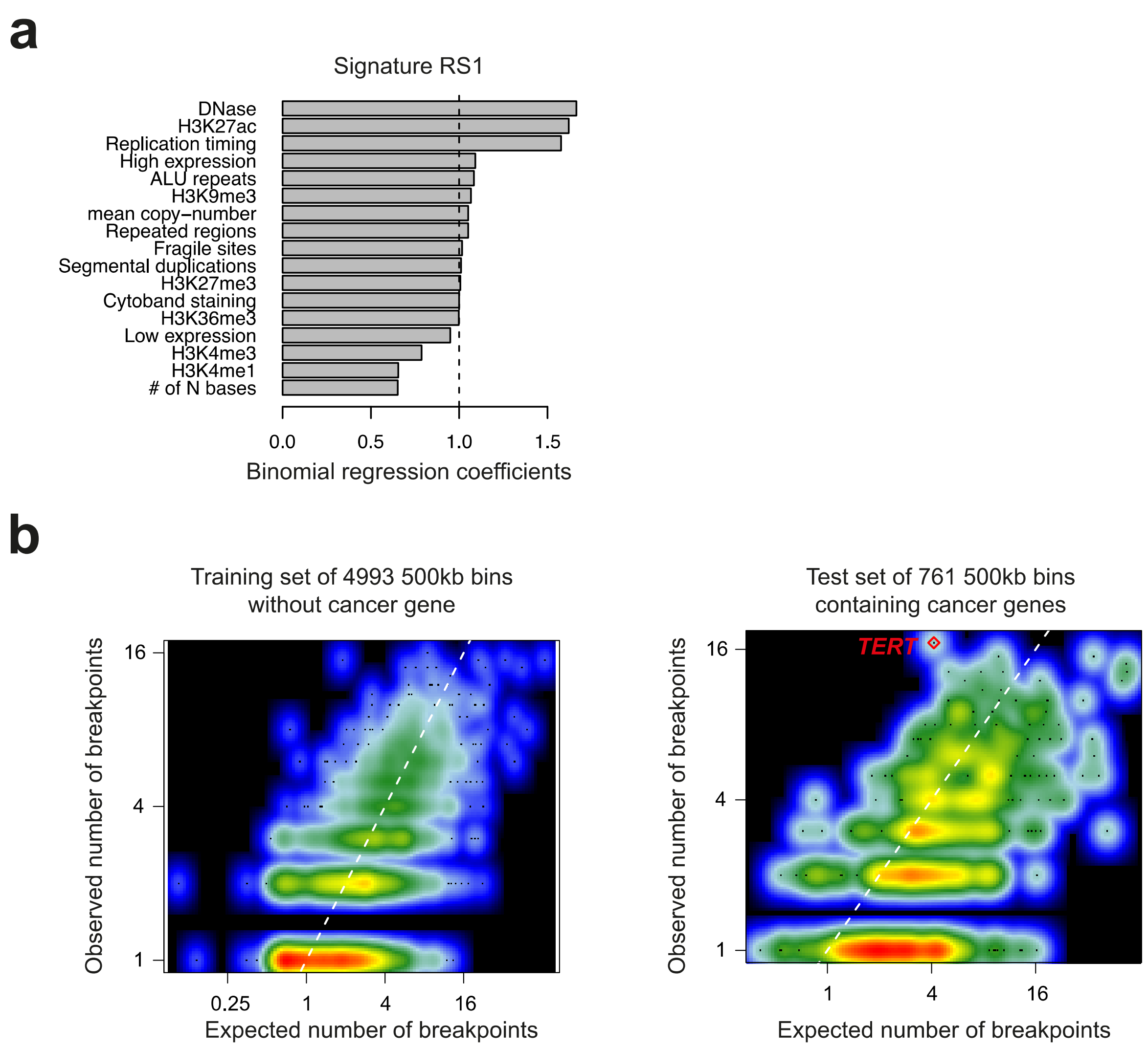

Supplementary Figure 9. Binomial regression modeling of rearrangement breakpoint density
a Regression coefficients of the 17 genomic features used to predict the density of signature RS1 breakpoints.
b Correlation between the number of observed RS1 breakpoints per 500 kb bin and the expected number predicted by the binomial regression model. Left: Within 4933 bins without any cancer gene used as training set. Right: Within 761 bins containing cancer genes (test set). The bin corresponding to *TERT* promoter region is highlighted in red.

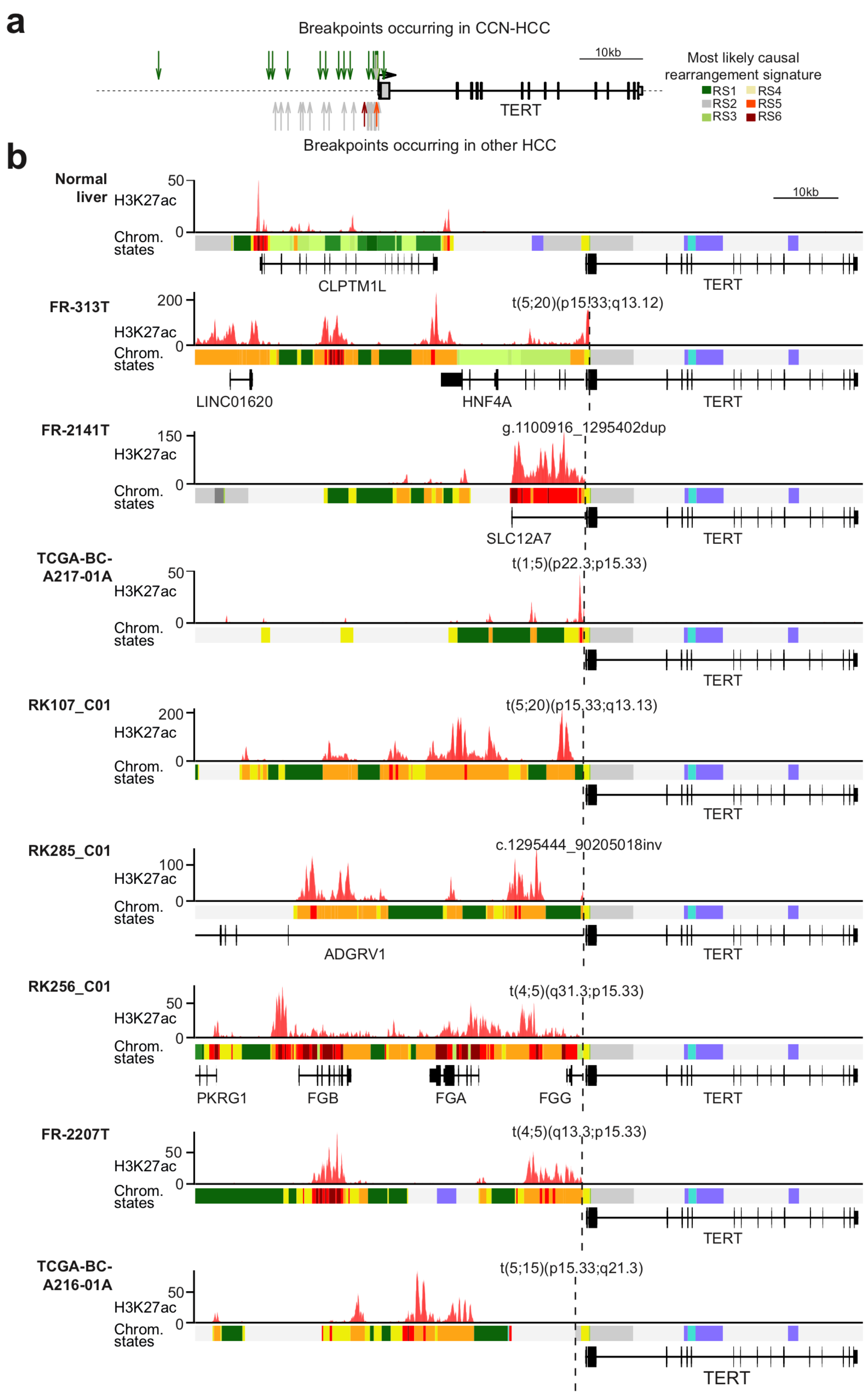
a
Breakpoints occurring in CCN-HCC
10kb
TERT
Most likely causal rearrangement signature
RS1
RS2
RS3
RS4
RS5
RS6
Breakpoints occurring in other HCC
b
Normal liver
H3K27ac
Chrom. states
10kb
CLPTM1L
TERT
FR-313T
t(5;20)(p15.33;q13.12)
LINC01620
HNF4A
FR-2141T
g.1100916_1295402dup
SLC12A7
TCGA-BC-A217-01A
t(1;5)(p22.3;p15.33)
RK107_C01
t(5;20)(p15.33;q13.13)
RK285_C01
c.1295444_90205018inv
ADGRV1
RK256_C01
t(4;5)(q31.3;p15.33)
PKRG1
FGB
FGA
FGG
FR-2207T
t(4;5)(q13.3;p15.33)
TCGA-BC-A216-01A
t(5;15)(p15.33;q21.3)

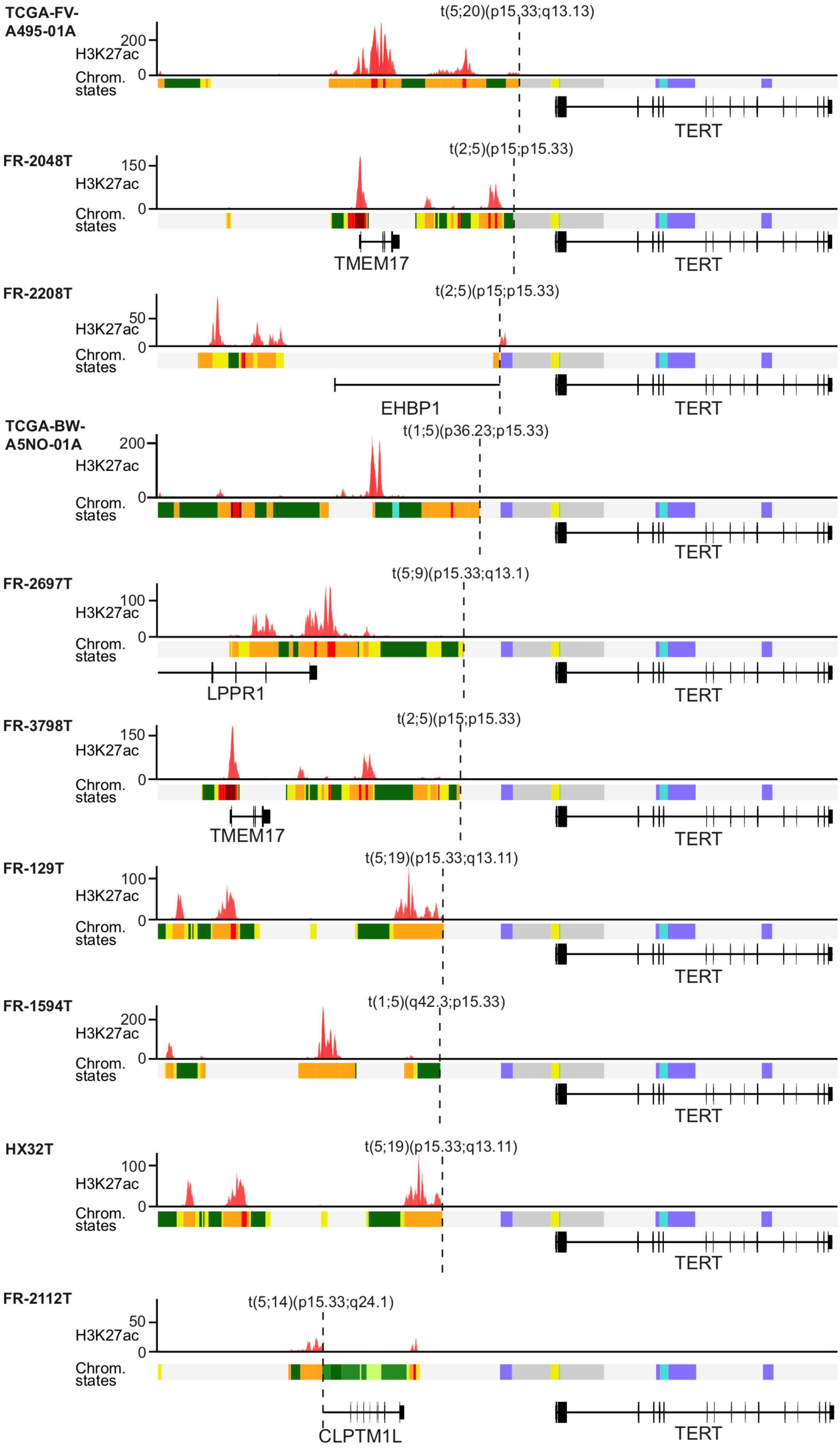
TCGA-FV-A495-01A
t(5;20)(p15.33;q13.13)
FR-2048T
t(2;5)(p15;p15.33)
TMEM17
FR-2208T
t(2;5)(p15;p15.33)
EHBP1
TCGA-BW-A5NO-01A
t(1;5)(p36.23;p15.33)
FR-2697T
t(5;9)(p15.33;q13.1)
LPPR1
FR-3798T
t(2;5)(p15;p15.33)
TMEM17
FR-129T
t(5;19)(p15.33;q13.11)
FR-1594T
t(1;5)(q42.3;p15.33)
HX32T
t(5;19)(p15.33;q13.11)
FR-2112T
t(5;14)(p15.33;q24.1)
CLPTM1L
H3K27ac
Chrom. states
TERT

Supplementary Figure 10. Rearrangements affecting *TERT* promoter region in 350 HCC genomes

a Summary of rearrangement breakpoints in *TERT* regulatory region. Each arrow indicates a rearrangement breakpoint. The color indicates the rearrangement signature of the most likely causal process. Breakpoints occurring in CCN-HCC are represented above the scheme. Those occurring in other HCC are represented below.

b Functional consequences of structural rearrangements affecting *TERT* regulatory region in CCN-HCC. Chromatin states and H3K27 acetylation in normal adult liver (top) are compared with predicted chromatin states and H3K27 acetylation resulting from the 18 structural rearrangements of *TERT* regulatory regions identified in CCN-HCC. H3K27Ac chromatin immunoprecipitation sequencing (ChIP-seq) signal were obtained from the ROADMAP consortium. The color code of chromatin states is the same as in **Supplementary Fig. 8**

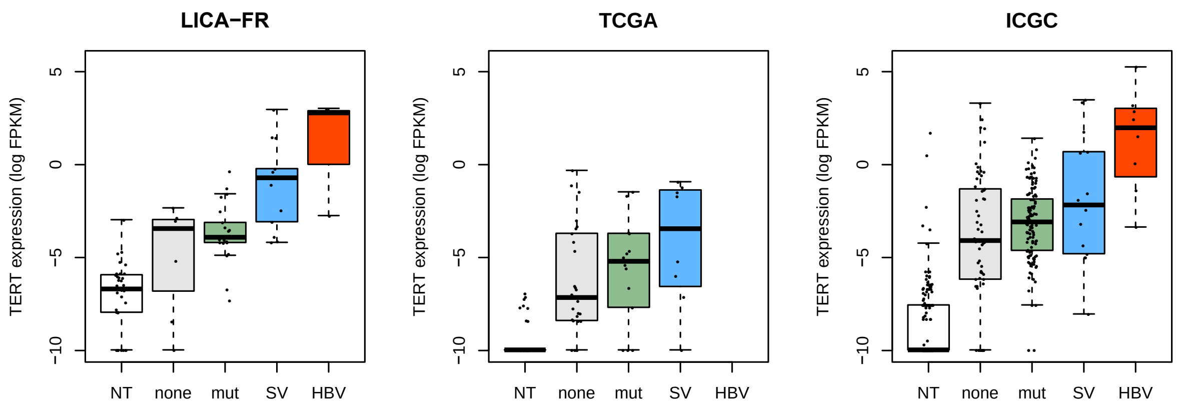

Supplementary Figure 11. Modulation of *TERT* expression by different types of genomic alterations.

The expression of *TERT* is shown in the 3 data sets as a function of the type of alteration identified in *TERT* promoter. Only samples with both whole genome and RNA sequencing were considered. No HBV insertion in *TERT* was identified among the 48 TCGA samples with whole genome sequencing.
NT: non-tumor liver sample; none: HCC without *TERT* alteration identified; mut: HCC with *TERT* promoter mutation; SV: HCC with structural rearrangement affecting *TERT* promoter; HBV: HCC with HBV insertion in *TERT* promoter. The middle bar, median; box, interquartile range; bars extend to 1.5 times the interquartile range.

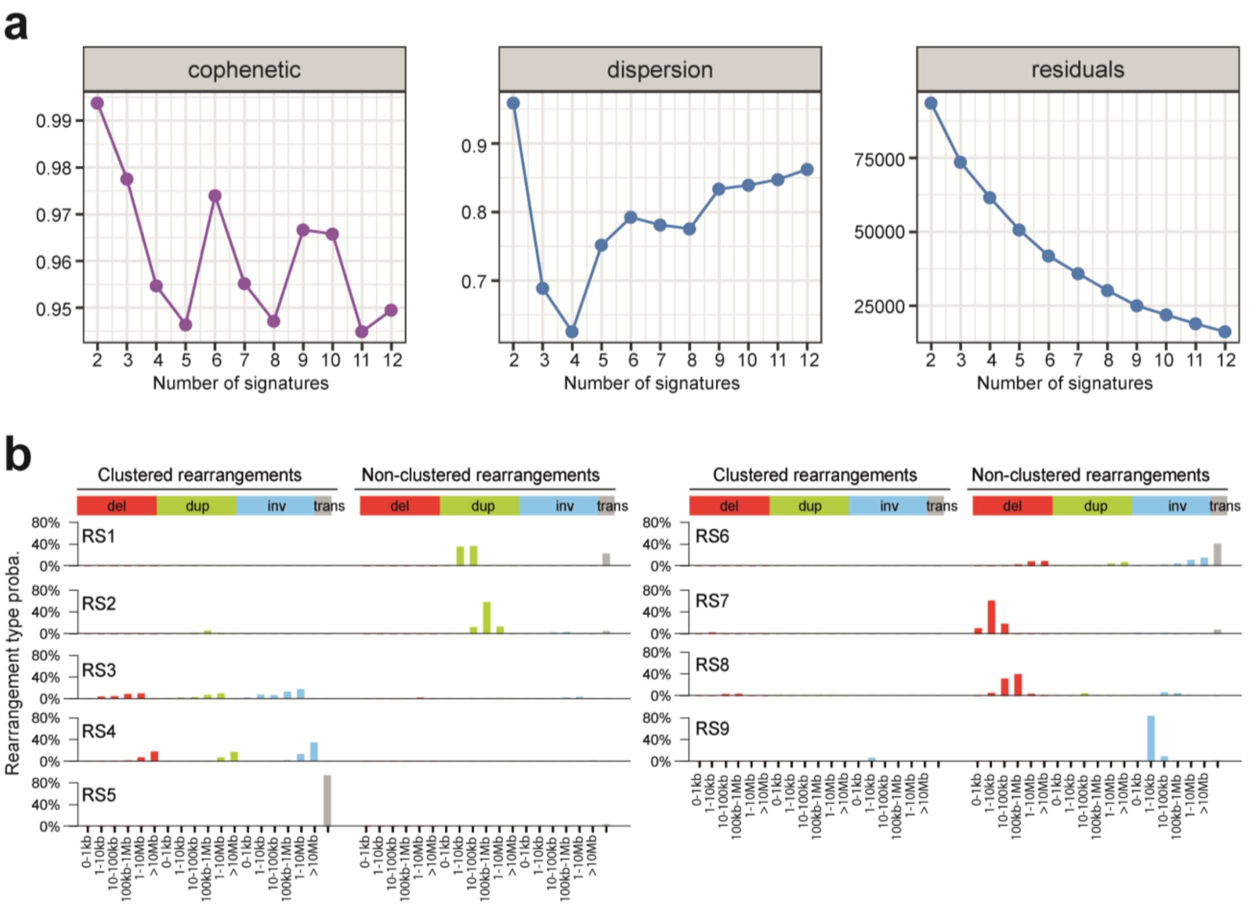

Supplementary Figure 12. Rearrangement signatures identified in the pan-cancer ICGC series

a Non-negative matrix factorization (NMF) metrics used to determine the optimal number of signatures. With 9 signatures, we obtain good cophenetic coefficient and dispersion score.

b Frequency of the 38 structural rearrangement categories in the 9 signatures.
