# Supplementary material for: Cyclin A2/E1 activation defines a hepatocellular carcinoma subclass with a rearrangement signature of replication stress
Source: Nat Commun. 2018 Dec 7;9:5235. doi: 10.1038/s41467-018-07552-9 (PMC6286353; doi:10.1038/s41467-018-07552-9)
Supplement: Supplementary file 2 — Description of Additional Supplementary Files [file 41467_2018_7552_MOESM2_ESM.docx]

**Description of Additional Supplementary Files**

**File Name**: Supplementary Data 1

**Description**: Clinical annotations for the 160 samples of the LICA-FR series

**File Name:** Supplementary Data 2

**Description**: Main clinical characteristics of the LICA-FR, TCGA and ICGC-JP cohorts

**File Name**: Supplementary Data 3

**Description**: Viral insertions identified at CCNA2 and CCNE1 loci

**File Name**: Supplementary Data 4

**Description**: Structural rearrangements identified at CCNA2 and CCNE1 loci

**File Name**: Supplementary Data 5

**Description**: Significantly de-regulated pathways in CCN-HCC

**File name**: Supplementary Data 6

**Description**: Significantly enriched and depleted driver genes in CCN-HCC

**File Name**: Supplementary Data 7

**Description**: Contribution of rearrangement signatures to the genomes of 350 tumors from the LICA-FR, TCGA and ICGC serie

**File Name**: Supplementary Data 8

**Description**: Rearrangements affecting TERT promoter region in 350 HCC genomes

**File Name:** Supplementary Data 9

**Description**: Number of RS1 events and association with CCNA2/E1 alterations across cancer types
